# Utilization of target lesion heterogeneity for treatment efficacy assessment in late stage lung cancer

**DOI:** 10.1371/journal.pone.0252041

**Published:** 2021-07-01

**Authors:** Dung-Tsa Chen, Wenyaw Chan, Zachary J. Thompson, Ram Thapa, Amer A. Beg, Andreas N. Saltos, Alberto A. Chiappori, Jhanelle E. Gray, Eric B. Haura, Trevor A. Rose, Ben Creelan

**Affiliations:** 1 Department of Biostatistics and Bioinformatics, H. Lee Moffitt Cancer Center & Research Institute, Tampa, Florida, United States of America; 2 Department of Biostatistics and Data Science, School of Public Health, University of Texas Health Science Center at Houston, Houston, Texas, United States of America; 3 Department of Immunotherapy, H. Lee Moffitt Cancer Center & Research Institute, Tampa, Florida, United States of America; 4 Department of Thoracic Oncology, H. Lee Moffitt Cancer Center & Research Institute, Tampa, Florida, United States of America; 5 Department of Radiology, H. Lee Moffitt Cancer Center & Research Institute, Tampa, Florida, United States of America; Sichuan University, CHINA

## Abstract

**Rationale:**

Recent studies have discovered several unique tumor response subgroups outside of response classification by Response Evaluation Criteria for Solid Tumors (RECIST), such as mixed response and oligometastasis. These subtypes have a distinctive property, *lesion heterogeneity* defined as diversity of tumor growth profiles in RECIST target lesions. Furthermore, many cancer clinical trials have been activated to evaluate various treatment options for heterogeneity-related subgroups (e.g., 29 trials so far listed in clinicaltrials.gov for cancer patients with oligometastasis). Some of the trials have shown survival benefit by tailored treatment strategies. This evidence presents the unmet need to incorporate lesion heterogeneity to improve RECIST response classification.

**Method:**

An approach for **Le**sion **He**terogeneity **C**lassification (LeHeC) was developed using a contemporary statistical approach to assess target lesion variation, characterize patient treatment response, and translate informative evidence to improving treatment strategy. A mixed effect linear model was used to determine lesion heterogeneity. Further analysis was conducted to classify various types of lesion variation and incorporate with RECIST to enhance response classification. A study cohort of 110 target lesions from 36 lung cancer patients was used for evaluation.

**Results:**

Due to small sample size issue, the result was exploratory in nature. By analyzing RECIST target lesion data, the LeHeC approach detected a high prevalence (n = 21; 58%) of lesion heterogeneity. Subgroup classification revealed several informative distinct subsets in a descending order of lesion heterogeneity: mix of progression and regression (n = 7), mix of progression and stability (n = 9), mix of regression and stability (n = 5), and non-heterogeneity (n = 15). Evaluation for association of lesion heterogeneity and RECIST best response classification showed lesion heterogeneity commonly occurred in each response group (stable disease: 16/27; 59%; partial response: 3/5; 60%; progression disease: 2/4; 50%). Survival analysis showed a differential trend of overall survival between heterogeneity and non-heterogeneity in RECIST response groups.

**Conclusion:**

This is the first study to evaluate lesion heterogeneity, an underappreciated metric, for RECIST application in oncology clinical trials. Results indicated lesion heterogeneity is not an uncommon event. The LeHeC approach could enhance RECIST response classification by utilizing granular lesion level discovery of heterogeneity.

## Background

### Rationale for incorporation of lesion heterogeneity to evaluate treatment responses in oncology clinical trials

As a standard tool to assess treatment efficacy in oncology clinical trials, Response Evaluation Criteria for Solid Tumors (RECIST) has helped advance cancer treatment, such as chemotherapy [1–4], targeted therapy [5–10], immunotherapy [11–17], or combinations of these [14, 15, 18–22]. In parallel, recent studies have discovered several unique tumor response subgroups outside of RECIST response classification, such as mixed response [23–26], oligometastasis [27–30], and pseudo-progression [31–33]. Patients in these subgroups often need special clinical attention to adapt treatment due to different reactions to the drugs of interest. These subtypes have a distinctive property, *lesion heterogeneity* defined as diversity of tumor growth profiles in RECIST target lesions. Moreover, some types of lesion heterogeneity were found to be associated with improved survival outcome by tailored treatment strategy [25, 27, 29, 34–38] in various cancers. For example, mixed responder to immunotherapy in metastatic melanoma had improved overall survival (OS) by surgical treatment [36]. Similarly, patients with oligometastatic lung or prostate cancer had longer progression-free survival or OS after stereotactic ablative radiation [27, 29, 35].

**Fig 1:**
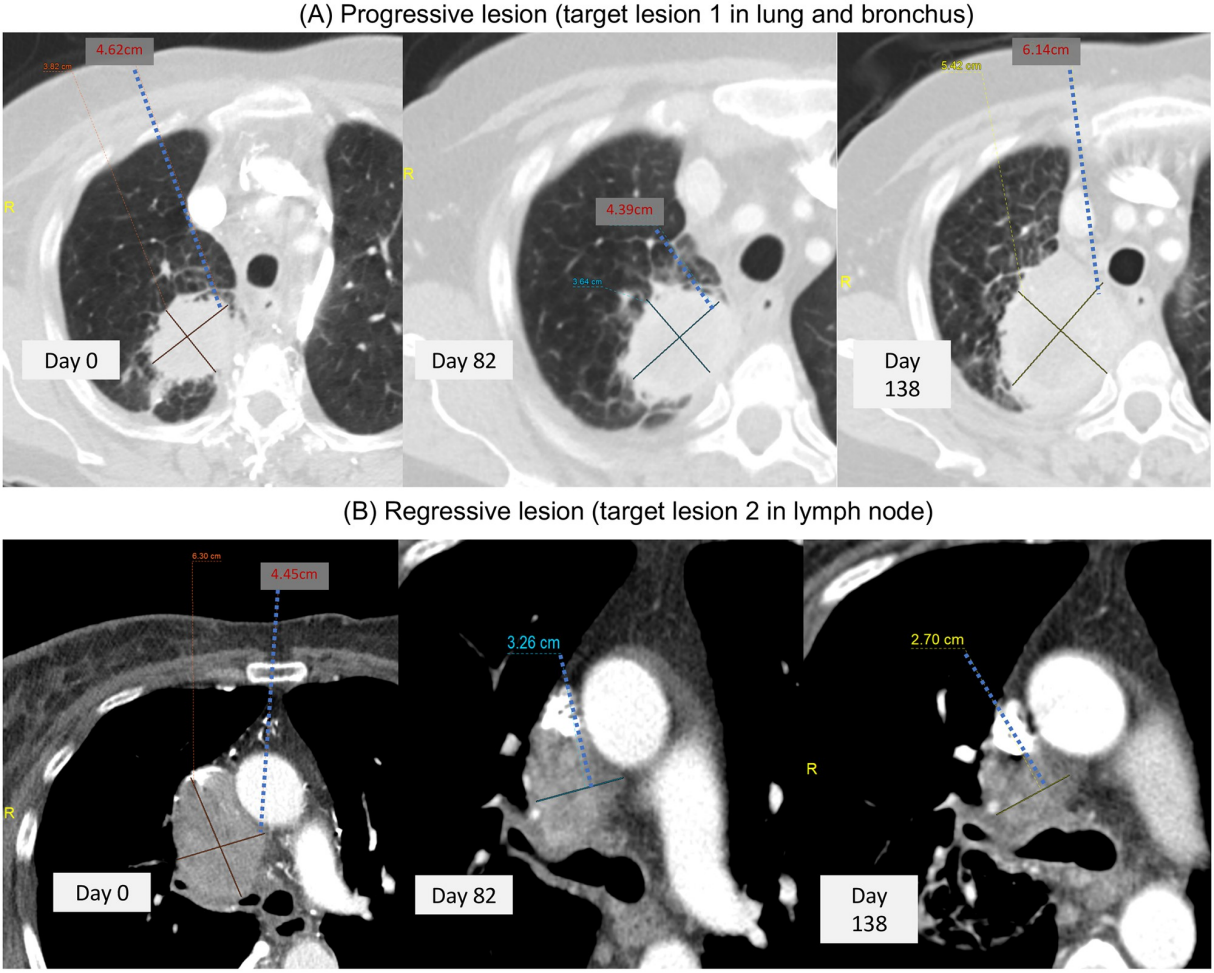
Lesion heterogeneity in CT image for Case A. (A): Illustrative image data of a progressive lesion for Case A. The lesion was target lesion 1 in lung and bronchus from three time points and demonstrated eventual progression of disease by RECIST criteria. The tumor growth pattern was also displayed in Fig 2A in black color dashed line. (B): Illustrative image data of a regressive lesion for Case A. The lesion was target lesion 2 in lymph node at three timepoints and showed continued decrease in size of lesion consistent with response to treatment. The tumor growth pattern was also displayed in Fig 2B in red color dashed line.

**Fig 2:**
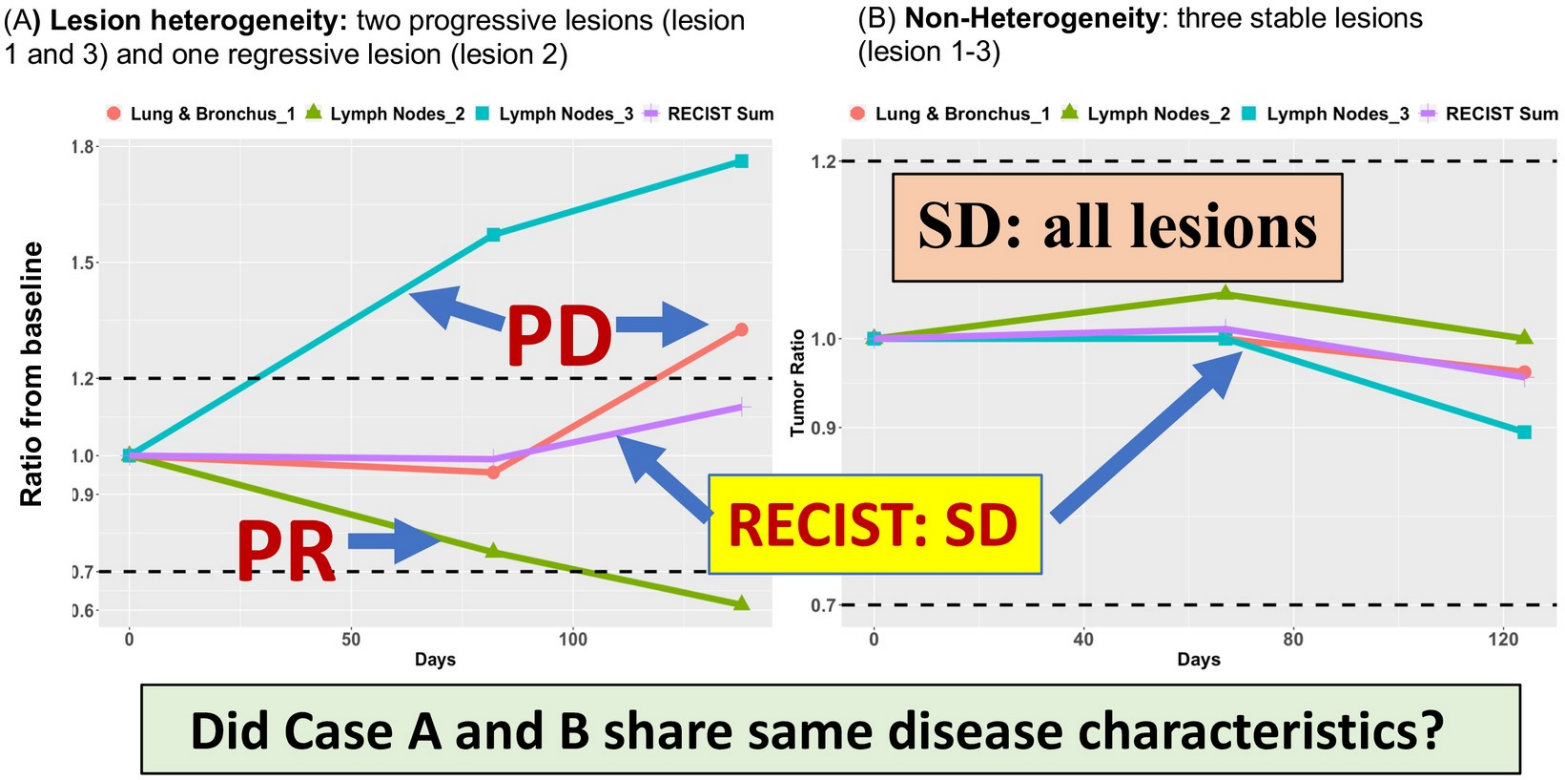
(A): Case A had three lesions with two in lymph node and one in lung site. In the lymph node site, one lesion had at least 30% reduction (ratio<0.7), but the other lesion progressed with more than 60% increase (ratio>1.6). The lesion in the lung site also increased the tumor size about 30%. Results showed completely different treatment reaction in each lesion with one regressive lesion and one progressive lesion in the lymph node and one progressive lesion in the lung site, indicating significant variation of lesion growth pattern within and between organ sites. However, the RECIST sum averaged these three heterogeneous lesions and classified as SD, suggesting all lesions were under control with no change of tumor growth. (B): In contrast, case B had also three lesions with two in lymph node and one in lung site. All lesion sizes remained relatively stable over time with minor changes from the baseline. The RECIST sum took an average of these three homogeneous lesions and also classified as SD. In comparison of both cases, while they reached the same SD classification, lesion variation to treatment reaction was quite different. Lesions in case B were comparable and did not progress or regress. On the other hand, case A had diverse tumor growth patterns composed of progressive and regressive lesions. While the RECIST sum might well characterize the stable condition in case B, classification of SD in case A seriously distorted lesion variation and could misguide clinical decision.

In addition, RECIST uses sum of all target lesions as overall tumor burden to determine treatment efficacy based on naïve assumption of lesion homogeneity. Such strategy significantly underestimates individual lesion variability of tumor growth [31, 32, 39, 40]. While many studies attempted to reform RECIST, their methodology still relied on RECIST’s aggregated sum to describe patient treatment response [41–44], including immune-related response criteria for immunotherapy trials (e.g., irRC [45], irRECIST [46], iRECIST [45], or imRECIST [33]). Figs 1 and 2 highlight limits of evaluating lesion heterogeneity by RECIST. Fig 1A and 1B showed CT images of two lesions with completely different response outcome in Case A: progression versus regression, indicating substantial variation of tumor growth among lesions. Fig 2 presented two contrastive cases with one in high degree of lesion heterogeneity (Case A with two progressive lesions and one regressive lesion: Fig 2A) and the other with homogenous lesions (Case B with three stable lesions: Fig 2B). RECIST classifies both cases as stable disease (SD). However, Case A showed opposite response among lesions, indicating potentially different disease and clinical characteristics from Case B. All these evidences (uniqueness of the heterogeneity, associated improvement of clinical outcomes, and limitations of RECIST) present the significant unmet need to incorporate lesion heterogeneity to improve RECIST.

## Methods

**Fig 3:**
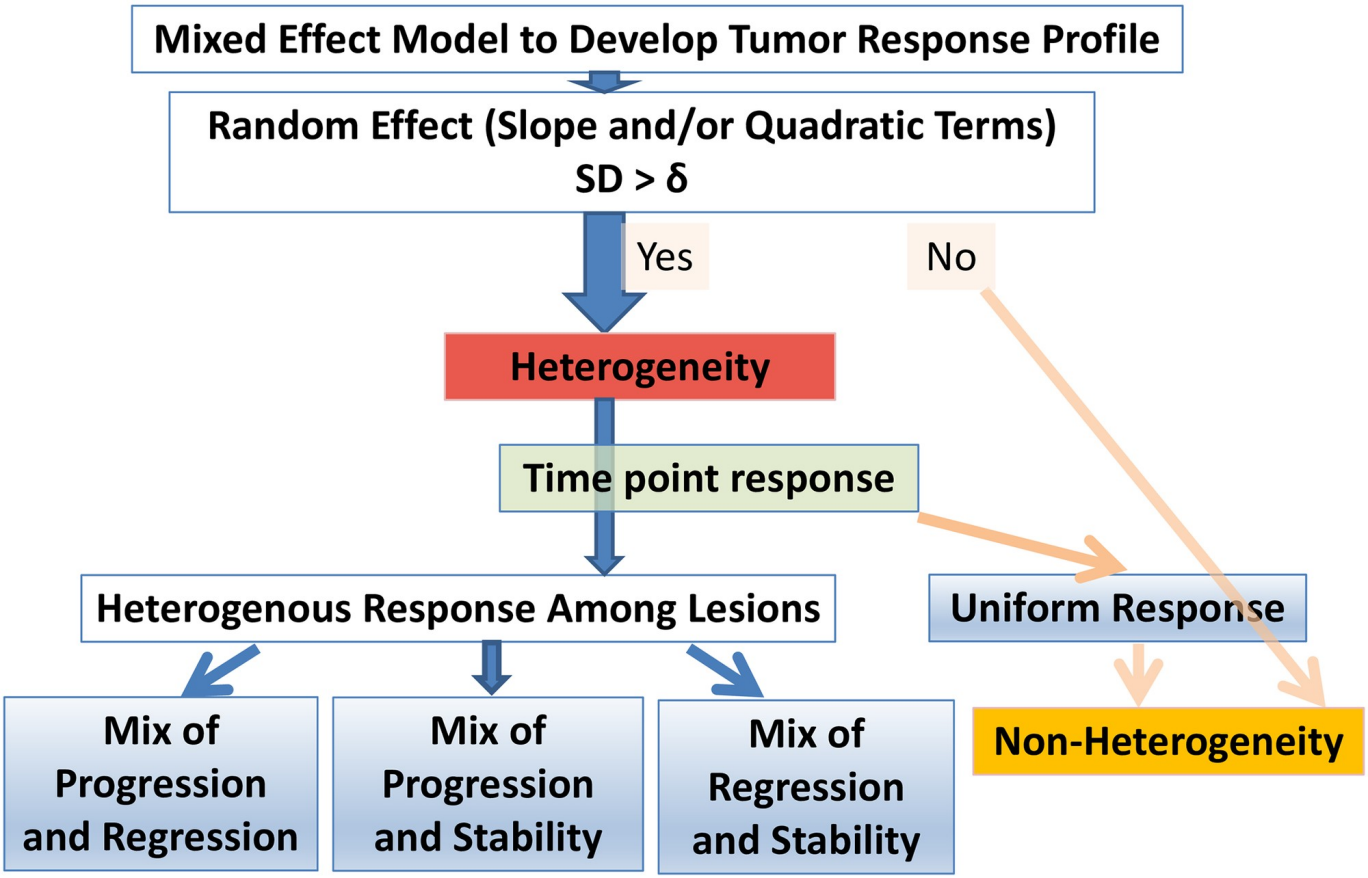
LeHeC algorithm.

This study takes advantage of target lesion level data, incorporates contemporary statistical method to assess lesion heterogeneity, and provides distinct subgroups to enrich RECIST response classification. Specifically, our approach for **Le**sion **He**terogeneity **C**lassification (LeHeC) utilizes multiple strategies to assess lesion heterogeneity (Fig 3): (1) mixed effect model to characterize tumor growth and determine lesion heterogeneity, (2) modified RECIST time point response to classify heterogeneity-associated subgroups, and (3) mean of squared deviations to evaluate classification performance.

### Mixed effect model for assessment of lesion heterogeneity

Statistically, a mixed effect regression model of lesion growths for a patient conducts two types of analysis: fixed effect and random effect. The fixed effect depicts overall tumor growth (mean function), similar to the RECIST sum metric. In contrast, the random effect component describes individual lesion’s deviation from the overall pattern and uses standard deviation (SD*) to summarize the deviation for growth parameters (e.g., linear slope or quadratic curve). Intuitively, when lesions grow diversely, the corresponding SD* will become large as shown in S1 Fig in S1 File. Therefore, SD* becomes a natural indicator of lesion heterogeneity. Strategically, lesion size from CT image is standardized and converted into ratio using the 1^st^ CT scan data as the baseline in each lesion. The logarithm of ratio is then used for analysis in the mixed effect model. The derived SD*s from the random components in linear slope or quadratic term are used to determine lesion heterogeneity (S1 Fig in S1 File). If a patient has the largest estimated SD*s above a threshold, δ, lesion heterogeneity is claimed initially.

### Modified RECIST time point response for subgroup classification

Once patients with lesion heterogeneity are identified (i.e., SD*>δ), they will be further classified based on characteristics of lesion variation using a modified RECIST response classification. Specifically, the modified RECIST response classification defines time point response as follows (S2 Fig in S1 File): (a) progression for a ratio > 1.2 (equivalent to 20% increase), (b) regression for a ratio <0.7 (equivalent to 30% reduction), and (c) stability for a ratio between 0.7 and 1.2. Each lesion thus has a set of time point responses to describe the degree of change in tumor size (e.g., stability in CT scan 1 and 2 and progression in CT scan 3). This strategy could address slow tumor progression issue [31] encountered in RECIST. Four heterogeneity-associated subgroups are defined based on comparison of time point responses among all lesions within a patient: (1) mix of progression and regression if lesions experience at least one event of progression and one event of regression. Mixed response and pseudo-progression are in this category because mixed response by definition is combination of progressive and regressive lesions while pseudo-progression has early progression event with follow-up of regression event due to treatment effect (e.g., immunotherapy); (2) mix of progression and stability if lesions experience at least one event of progression and one event of stability, and without regression event. Oligometastasis belongs to this category because of its unique property with majority of stable lesions and few progressive lesions; (3) mix of regression and stability if lesions develop at least one event of regression and one event of stability, and without progression event; and (4) non-heterogeneity if all lesions had stability event. Patients with SD*<δ are also considered as non-heterogeneity.

### Mean of squared deviations (MSD) for classification performance

A set of MSD metrics are used to evaluate classification performance for the LeHeC approach in three aspects, deviation from RECIST sum, outlier detection, and model goodness of fit.

Deviation from RECIST sum (S3 Fig in S1 File): When lesion growth patterns within a patient are largely different from the RECIST sum, degree of lesion heterogeneity will be high accordingly. Thus, deviation from the RECIST sum could be a natural metric to evaluate classification performance for lesion heterogeneity. Our strategy is to compare log ratio of each lesion to the one in the RECIST sum. Difference between lesions and the RECIST sum will be squared and averaged, denoted as *MSD*_*(RECIST)*_, to assess magnitude of lesion heterogeneity. We expect the largest *MSD*_*(RECIST)*_ in patients with a mix of progressive and regressive lesions, followed by the subgroup with a mix of progressive and stable lesions, the subgroup with mixed lesions of regression and stability, and the non-heterogeneity subgroup in a descending order of *MSD*_*(RECIST)*_. We also evaluate *MSD* in the mixed effect model by measuring deviation away from the mean function and denoted as *MSD*_*(model)*_. We consider that the mean function of the mixed effect model is able to resemble the RECIST sum and expect *MSD*_*(model)*_ to be comparable to *MSD*_*(RECIST)*_.

Detection of outlier lesion (S4 Fig in S1 File): Another metric to examine lesion heterogeneity is number of lesions with substantial deviation from the model mean function (i.e., frequency of outlier lesions). A lesion level of mean squared deviations, *MSD*_*(model_lesion)*_, is used to determine outlier lesion. It compares log ratio between a lesion and the model mean function at each time point. The difference is squared and averaged, then defined as *MSD*_*(model_lesion)*_. A large value of *MSD*_*(model_lesion)*_ indicates the lesion has a different tumor growth pattern and will be considered as an outlier lesion if it exceeds a cutoff, **γ**.

Model goodness of fit (S5 Fig in S1 File): To ensure the model fits well within the lesion level data in the heterogeneity-associated subgroups, a quasi *R*^*2*^ is used to measure goodness of fit and defined as RMSD2=1-MSDlesionMSDmodel where *MSD*_*(lesion)*_ is calculated by the mean of squared differences across each time between predicted lesion curves and the observed lesion curves in a patient. A value of $R_{MSD}^{2}$ close to 1 indicates good fit of data while a near 0 value implies poor prediction.

### Statistical analysis

Evaluation of lesion heterogeneity was performed by two-sample t test, one-way analysis of variance (ANOVA), and Dunnett test to compare the heterogeneity group or subgroups to the non-heterogeneity group. Survival curve was generated by Kaplan-Meier (KM) method. The log-rank test was then used to evaluate survival difference due to heterogeneity in each RECIST response group.

### Study cohort

This retrospective study was derived from a research protocol (MCC20369 approved by Scientific Review Committee and IRB exempt) at the Moffitt Cancer Center and Research Institute. Data used for the study was from 36 late stage lung cancer patients receiving immunotherapy at the institute between October 2015 and July 2018. The demographic characteristics of patients were presented in Table 1. Distribution of RECIST best overall response was 5 partial responses (PR), 27 stable diseases (SD), and 4 progression diseases (PD). A total of 110 target lesions from the 36 patients were collected. Tumor measurements of these lesions were calculated per RECIST from CT scans for data analysis. Distribution of CT scans were 50% with 3 scans (n = 18), and 50% with 4 scans or more (n = 18). For lesion frequency per patient, there were 33% patients with 2 lesions (n = 12), 33% patients with 3 lesions (n = 12), 28% patients with 4 lesions (n = 10), and 6% patients with 5 lesions (n = 2). Data and R code were provided in S1 File.

**Table 1:** Demographic characteristics of 36 patients with stage 4 NSCLC.

Baseline and clinical variable		N (%)
**Gender**	Female	15 (42%)
	Male	21 (58%)
**Race/Ethnicity**	Non-Hispanic White	28 (78%)
	Hispanic	4 (11%)
	Black	2 (6%)
	Asian	1 (3%)
	Unknown	1 (3%)
**Smoking history**	Current smoker	2 (6%)
	Previous smoker	11 (31%)
	Never	4 (11%)
	Unknown	19 (53%)
**Best overall response**	PR	5 (14%)
	SD	27 (75%)
	PD	4 (11%)
**OS**	Dead	28 (78%)
	Alive	8 (22%)
	Median (range)	
**Follow-up time**	10.41 months (2.01–36.26)	
**Age**	64 (38–80)	

## Results

### LeHeC classification associated with lesion heterogeneity

**Fig 4:**
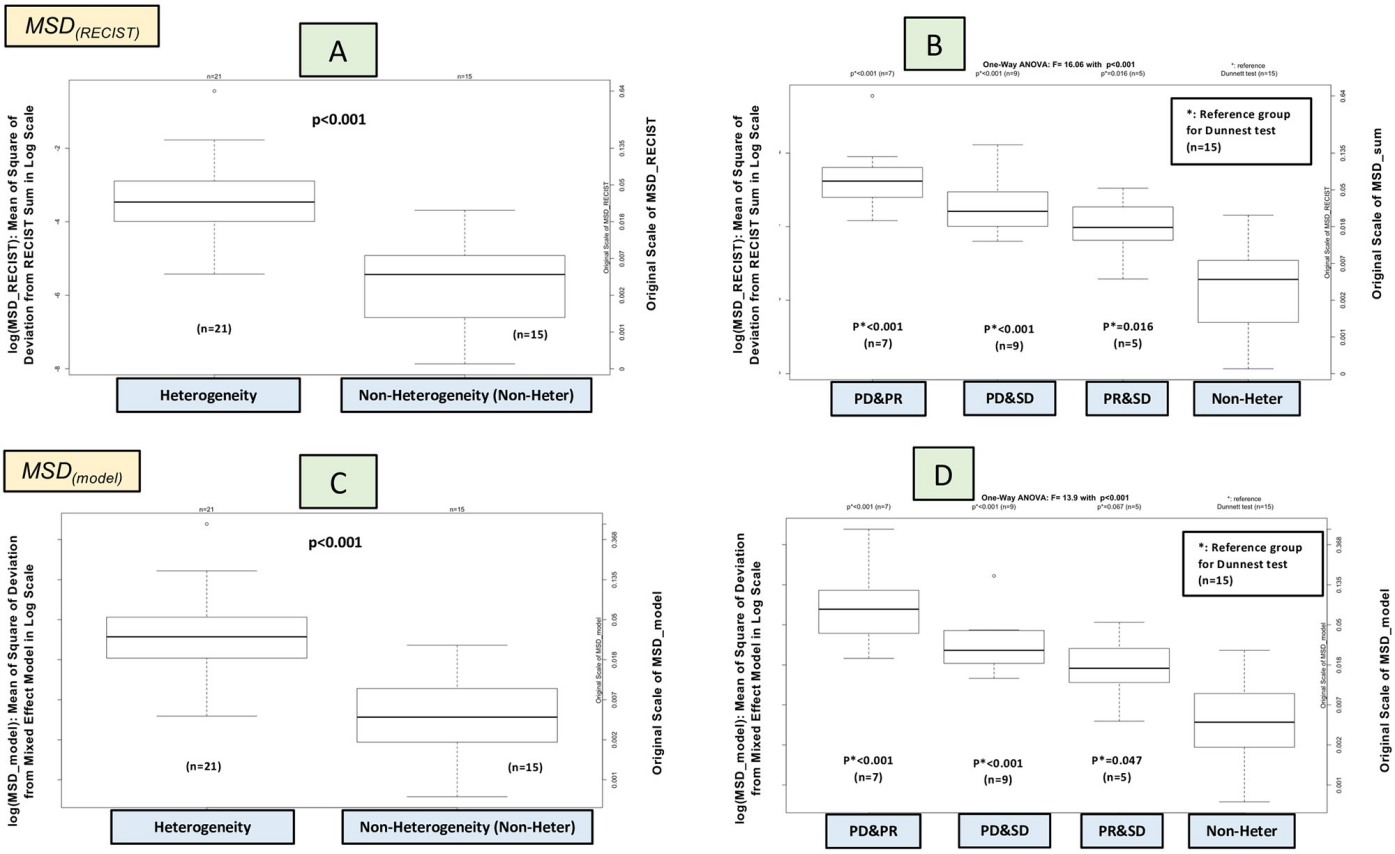
Comparison of MSD among lesion heterogeneous subgroups. **PD&PR**: mix of progression and regression; **PD&SD**: mix of regression and stability; **PR&SD**: mix of regression and stability; **Non-Heter**: non-heterogeneity.

The LeHeC approach identified 21 patients (58%) with heterogeneous lesions and 15 patients (42%) with non-heterogeneity based on a threshold δ = 0.15 for SD*. Determination of the threshold was based on empirical evaluation of change in slope. which occurred at 0.15 (S6A Fig in S1 File). The lesion heterogeneity-associated group showed a significantly higher *MSD*_*(RECIST)*_ (p<0.001 by t-test; Fig 4A). Further subgroup analysis unveiled a decreasing trend of median of log(*MSD*_*(RECIST)*_) with the highest in the mix of progression and regression (n = 7; 19%), followed by the mix of progression and stability (n = 9; 25%), then by the mix of regression and stability (n = 5; 14%), and lastly by the non-heterogeneity (n = 15; 42%). One-way ANOVA and Dunnett test showed an overall group difference (p<0.001; Fig 4B) and subgroup difference between the first three subsets and the non-heterogeneity group (p<0.05; Fig 4B). Analysis of *MSD*_*(model)*_ for the mixed effect model also yielded comparable results in evaluation of lesion heterogeneity (Fig 4C and 4D and S6 Fig in S1 File). The order of lesion heterogeneity was further assured by analysis of outlier lesion based on a cutoff γ = 0.045 derived from the 80^th^ percentile *MSD*_*(model_lesion)*_ among the 110 lesions. The approach identified most outlier lesions in the mix of progression and regression (12 lesions), 7 outliers in the mix of progression and stability, and 3 outliers in the mix of regression and stability, and no outlier lesions detected in the non-heterogeneity group (S7 Fig in S1 File). The random effect model had 71% of $R_{MSD}^{2}$>0.8 (S8 Fig in S1 File: percentile of 25^th^, 50^th^, 75^th^: 0.79, 0.90, 0.94). The high $R_{MSD}^{2}$ value indicates the predicted lesion curves by the random effect model were able to depict the observed lesion curves. Distribution of $R_{MSD}^{2}$ was comparable with a median value>0.8 among the 3 heterogeneity-associated subgroups (p = 0.91 by one-way ANOVA). All these results showed that the classification was able to differentiate lesion heterogeneity.

### Enrichment of RECIST lesion-heterogeneity response classification

The 4 subgroups were detailed in each RECIST response category. Overall, lesion heterogeneity commonly occurred in each RECIST response group (Table 2).

**Table 2:** Frequency of the heterogeneity associated subgroups in each RECIST response category.

RECIST (Best Response)	Mix of Progression and Regression	Mix of Progression and Stability	Mix of Regression and Stability	Non-Heterogeneity
Stable Disease (SD)	5	8	3	11
Partial Response (PR)	2	0	1	2
Progressive Disease (PD)	0	1	1	2

#### RECIST SD (n = 27)

**Fig 5:**
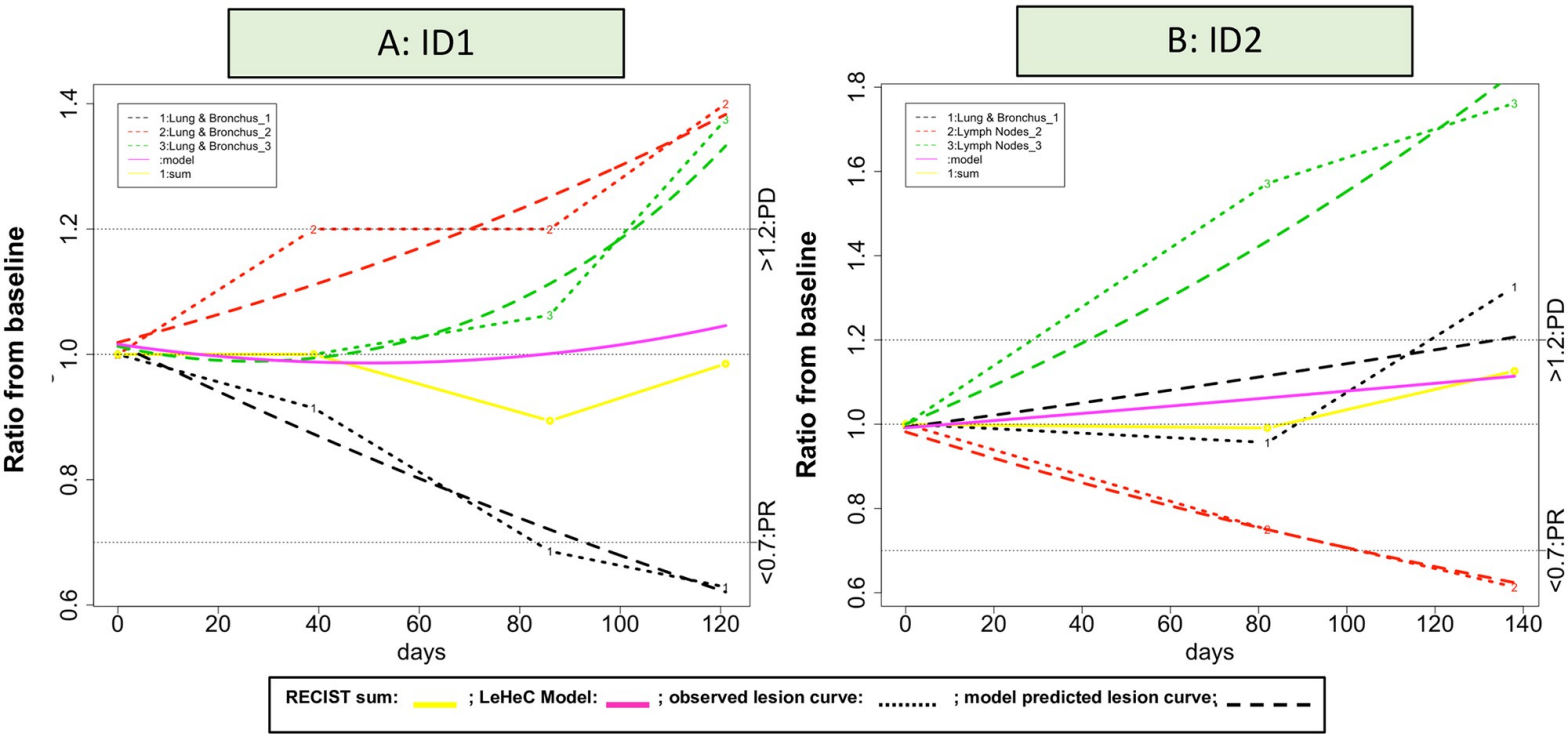
Mixed response.

This group had 16 cases (59%) with lesion heterogeneity. Specifically, five were classified as a mix of progression and regression. Two cases had a clear diverse pattern with more than 30% reduction in one lesion and over 20% increase in other lesions over time (Fig 5: ID 1–2). The other three cases showed a sporadic mixed response defined as some stable lesions with temporary regression at some time points or some lesions experienced regression to progression over time (S9 Fig in S1 File: ID S9 1–3). Eight cases showed a mix of progression and stability with three subtle patterns (S10 Fig in S1 File). One had most stable lesions with a near flat growth rate, but had only one or few progressive lesions with a high growth rate (n = 3; S10A Fig in S1 File: ID S10A 1–3). Such pattern is relevant to oligometastasis. Another pattern had most progressive lesions and few stable lesions (n = 1; S10B Fig in S1 File; ID S10B-1). The other pattern was a balanced mix of progressive and stable lesions (n = 4; S10B Fig; ID S10B 2, 4–6). Three cases showed a mix of regression and stability (S11 Fig in S1 File; ID S11A2 and B1-2). Their regressive lesions had either tumor reduction over time or temporary regression. Eleven cases did not show lesion heterogeneity (S12 Fig in S1 File). They had similar response profiles among lesions either in stable condition (n = 8; S12A Fig; ID S12A 1–7, 9) or consistent growth curve in an increasing, decreasing, or irregular trend (n = 3; S12B Fig in S1 File; ID S12B 1–3).

#### RECIST PR (n = 5)

**Fig 6:**
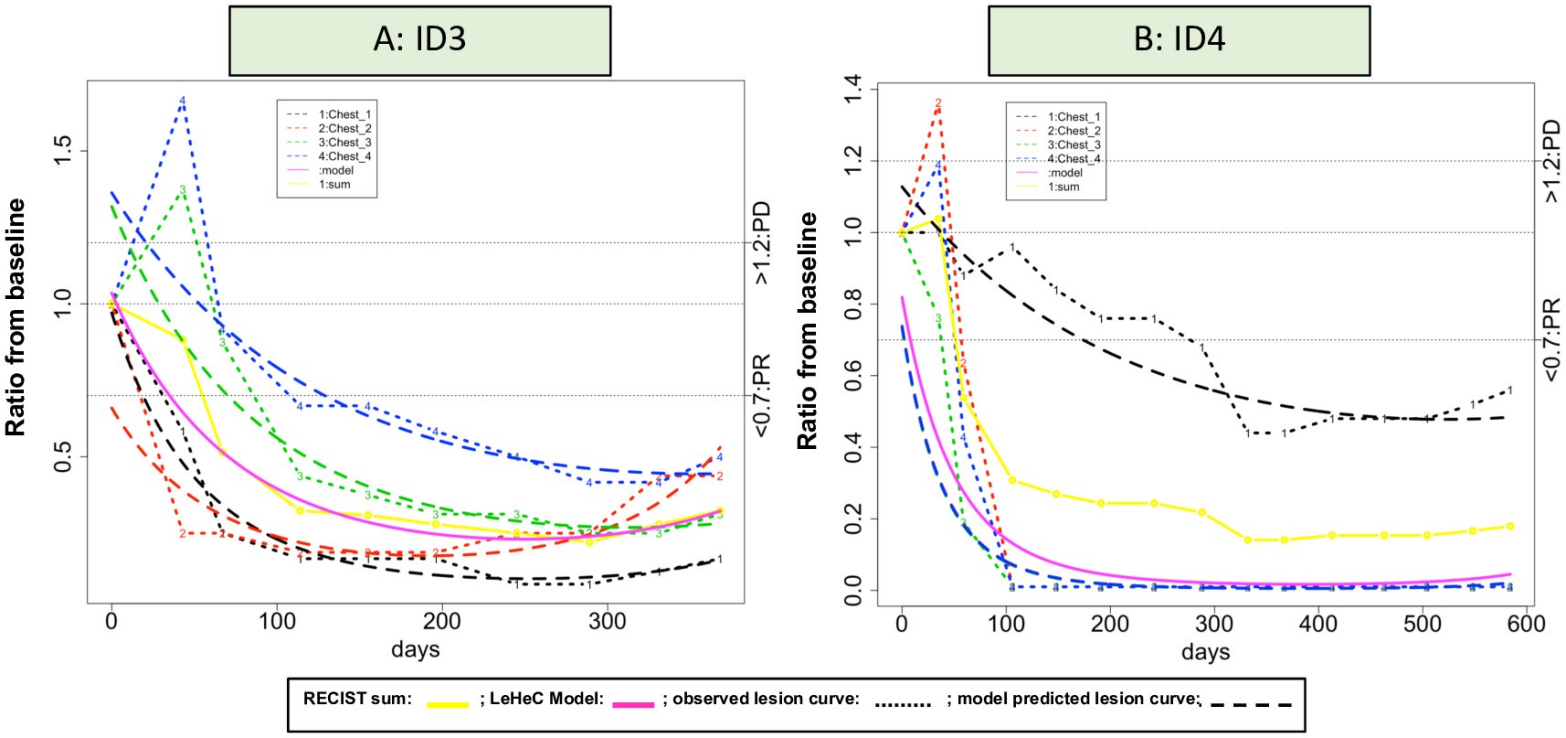
Mix of pseudo-progressive and regressive lesions. (A): Case ID 3 had 4 chest lesions with two chest lesions (lesion 3-4) experienced early progression and then developed response with a ratio below 0.7 after day 100. The other two chest lesions had a ratio below 0.7 after the 1^st^ CT scan. (B): Case ID 4 had a similar pattern where an initial ratio >1.2 and then disappeared after 100 days in two upper lobe nodule lesions (lesion 2 and 4). The two lower lobe nodule lesions had tumor reduction at least 30% with one after day 100 and the other one after day 300.

Two had a mix of progression and regression (Fig 6). The progression part was caused by pseudo-progressive lesions with initial significant increase and later substantial reduction of tumor size. One case with a mix of regression and stability had two lesions with initial regressive trend, but later split into one stable lesion and one regressive lesion (S11B Fig in S1 File; ID S11B3). The two cases with non-heterogeneity had either with homogenous regressive lesions or a pattern with initial decline and later growth (S12B Fig in S1 File; ID S12B 5–6). Notably, the three heterogeneity cases had a longer duration of treatment over 300 days, compared to the two cases without heterogeneity (<120 days).

#### RECIST PD (n = 4)

PD was determined due to early new lesion (n = 3) or unequivocal increase of non-target lesion (n = 1). One had a mix of progression and stability (S10B Fig in S1 File; ID S10B3), one had a mix of regression and stability (S11A Fig in S1 File; ID S11A1), and two had non-heterogeneity (S12 Fig in S1 File; ID S12 A8 and B4).

### Survival analysis

**Fig 7:**
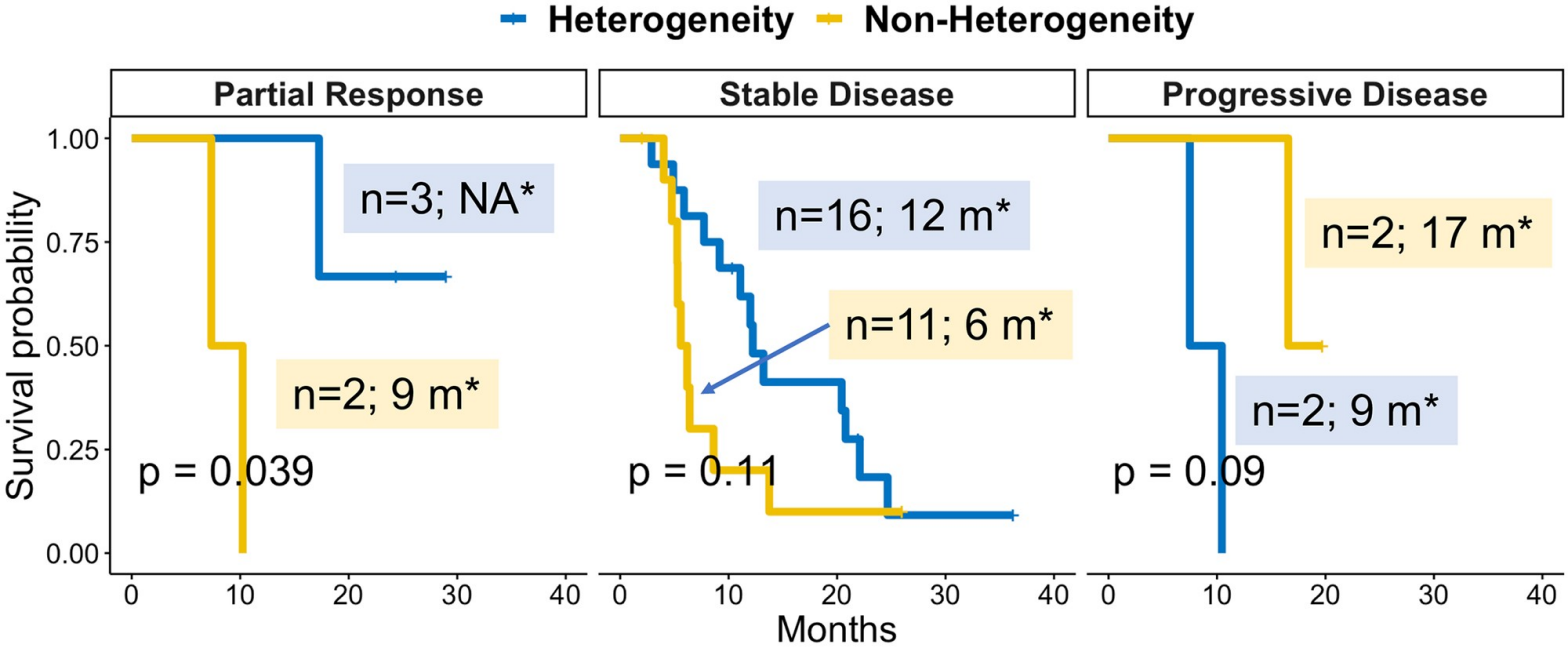
Survival analysis. *: median OS; m: month.

Survival analysis was conducted in each RECIST response group (PR, SD, and PD). Results (Fig 7) showed the heterogeneity group with a longer OS in patients with PR and SD, but a shorter OS in patients with PD. Specifically, for PR (n = 4), the median OS was not reached in the heterogeneity subgroup (n = 3) and 9 months in the non-heterogeneity subgroup (n = 2) (p = 0.039). The SD group also had a similar trend with a median OS of 12 and 6 months in the heterogeneity (n = 16) and non-heterogeneity (n = 11) subgroups, respectively (p = 0.11). The PD showed a different pattern with a median OS of 9 and 17 months in the heterogeneity (n = 2) and non-heterogeneity (n = 2) subgroups, respectively (p = 0.09).

## Discussion

This is the first study to utilize target lesion data to assess lesion heterogeneity. The results support evidence of high prevalence of lesion heterogeneity in cancer patients (~20% mixed response [24, 36, 47], 20–50% oligometastasis [48], and 5–36% pseudo-progression [39, 49]). Moreover, analysis by the LeHeC approach showed granular lesion level discovery of heterogeneity to enrich RECIST response classification.

Methodology-wise, the LeHeC approach first employs the mixed effect model to depict tumor growth patterns and takes advantage of random effect components to quantify lesion heterogeneity. The modified RECIST time point response is then applied to classify four distinct subgroups with potential clinical application. Moreover, a set of diagnosis tools using various forms of MSD is deployed to examine classification performance. Specifically, the LeHeC approach identified more than 50% patients with heterogeneous lesions by assessing variation of lesion growth curve parameters through SD*. Lesion deviation from the RECIST sum and the mixed effect mean function (*MSD*_*(RECIST)*_ and *MSD*_*(model)*_) validated the classification result by showing a significantly larger deviation in patients with lesion heterogeneity. Subgroup classification by the modified RECIST time point response further formed 4 informative subsets in a descending order of lesion heterogeneity: mix of progression and regression, mix of progression and stability, mix of regression and stability, and non-heterogeneity. The pattern was also supported by lesion deviation from *MSD*_*(RECIST)*_ and *MSD*_*(model)*_, as well as analysis of outlier lesion detection. Moreover, 71% cases had $R_{MSD}^{2}$>0.8 supporting a high degree of model goodness of fit by the mixed effect model in predicting observed lesion curves.

From clinical prospective, significant efforts have been made in clinical trials to determine treatment effect. However, utilization of target lesion data has been suboptimal by the RECIST sum metric, especially in abundance of lesion heterogeneity as shown in this study and literature [24, 36, 39, 47–49]. Subgroup classification by the LeHeC approach revealed the 4 distinct heterogeneity-associated subgroups. The heterogeneity-associated subgroups did not show association with RECIST best response groups (p = 0.63; Table 2). Instead, lesion heterogeneity was pervasive (50–60%) in each response group (SD: 16/27; 59%; PR: 3/5; 60%; PD: 2/4; 50%).

Specifically, for patients with SD, they experienced all the 4 heterogeneity-associate subtypes, indicating attention needed for lesion heterogeneity. In particular, the mix of progression and regression identified two mixed responders with some progressive lesions and some regressive lesions (Fig 5). The RECIST took the sum, consequentially cancelled out both opposite treatment effects, and presented it as SD. The LeHeC approach realized the issue and incorporated such heterogeneity information into RECIST so clinicians could have better knowledge to determine if “lesionalized” tailored treatment strategy is needed. In fact, several studies [25, 50, 51] noticed mixed response and reported clinical benefit in some mixed responders using personalized treatment. The mix of progression and stability also identified a unique subtype, combination of a few progressive lesions with majority of stable lesions, which resembles oligometastasis. Since oligometastasis is clinically actionable (e.g., stereotactic radiation, cryoablation, and minimally invasive surgery; 29 oligometastasis-associated trials listed in clinicaltrials.gov so far), this subtype may deserve attention for potential tailored treatment strategy. Patients with PR had two cases with pseudo-progressive lesions and regressive lesions in the mix of progression and regression. Interestingly, they had a longer duration of treatment over 12 months. While a study [52] showed the survival benefit of pseudo-progression over progression in patients with PD, the hypothesis of a clinical benefit by pseudo-progressive lesions over regressive lesions may be worth the investigation.

Moreover, survival analysis showed some interesting results: heterogeneity group had a longer OS in patients with PR and SD, but a shorter OS in patients with PD. The last part was consistent with literature considering heterogeneity as poor prognosis, such as mixed response [24]. In contrast, the first part was a new discovery that heterogeneity improves OS for non-PD patients, with p<0.05 for PR patients. It also showed an interaction pattern of lesion heterogeneity and RECIST classification in predicting OS, meaning heterogeneity affected OS differently among RECIST response groups. Nevertheless, due to the small sample size issue, the results were preliminary.

The study has several limitations. The major concern is the small sample size from a single institution which has limited various analysis. One issue is to restrict the study into exploratory nature without confirmatory results. However, even with this constraint, the study was able to support lesion heterogeneity as a common event and revealed distinct heterogeneity subtypes to tune up RECIST classification. Another issue is lack of power for survival analysis to test if the new discovery heterogeneity-associated subtypes could predict OS (prognostic biomarker) and/or treatment (predictive biomarker). Even so with this small sample size, the study showed a differential OS trend of heterogeneity versus non-heterogeneity from PR, SD, to PD (Fig 7). The other limitation is small frequency of heterogeneity subtypes. It raises uncertainty whether some subtypes of lesion heterogeneity were due to randomness. Future multiple large retrospective studies across multiple institutes and prospective randomized interventional trials are needed to address these concerns and draw definitive conclusion.

In summary, this study raises awareness of the heterogeneity issue, quantitatively assesses lesion variation, and utilizes the heterogeneity information to tune up RECIST response classification. These contributions are valuable since this research field is still at infancy stage. Moreover, the concept of lesion heterogeneity fits naturally into principle of precision medicine by the potential application of lesion-guided treatment strategy. Thus, incorporation of this component could enhance RECIST assessment of treatment efficacy in granular lesion level. Moreover, while our current research is limited to the patients experienced with PD, or the ones with completion of treatment, the LeHeC approach has potential to guide treatment management in two scenarios: (a) SD case: during clinical trial, patient will continue treatment if overall tumor burden shows SD at current tumor measurement (i.e., CT scan). However, the SD could mean a result of either homogenous stable lesions or a mix of progressive and regressive lesions. The LeHeC approach could help differentiate both patterns. Such information could help clinicians determine if adaptive treatment strategy is needed for the latter case before it reaches PD or completion of treatment, (b) mix of pseudo-progressive and regressive lesions: Two cases were observed with more than one year of treatment duration. While this observation is limited due to small sample size, it prompts the question of whether such lesion heterogeneity has a longer response duration than the one with homogenous regressive lesions. If the hypothesis is true, the LeHeC approach could help early detect such a mix of pseudo-progressive and regressive lesions so clinicians could adjust treatment strategy accordingly.

## Supporting information

S1 File R code for lesion heterogeneity, and data of lesion heterogeneity. (PDF)
