# Correction: Utilization of target lesion heterogeneity for treatment efficacy assessment in late stage lung cancer

**DOI:** 10.1371/journal.pone.0255429

**Published:** 2021-07-23

**Authors:** The PLOS ONE Staff

The statement in the Funding section incorrectly reproduces the Competing Interests statement. The publisher apologizes for the error. The correct Funding statement is: Support for this study included departmental funds from the Department of Biostatistics and Bioinformatics, the Biostatistics and Bioinformatics Shared Resource, and Lung Cancer Center of Excellence at the H. Lee Moffitt Cancer Center & Research Institute, Stand Up To Cancer (SU2C-AACR-CT04-17), and the National Institutes of Health (5P30CA076292).
