# Supplementary material for: Utilization of target lesion heterogeneity for treatment efficacy assessment in late stage lung cancer
Source: PLoS One. 2021 Jul 1;16(7):e0252041. doi: 10.1371/journal.pone.0252041 (PMC8248740; doi:10.1371/journal.pone.0252041)
Supplement: S1 File — (PDF) [file pone.0252041.s001.pdf]

S1 Fig. Standard deviation (SD*) of random effect

Figure S1: Illustration of SD*

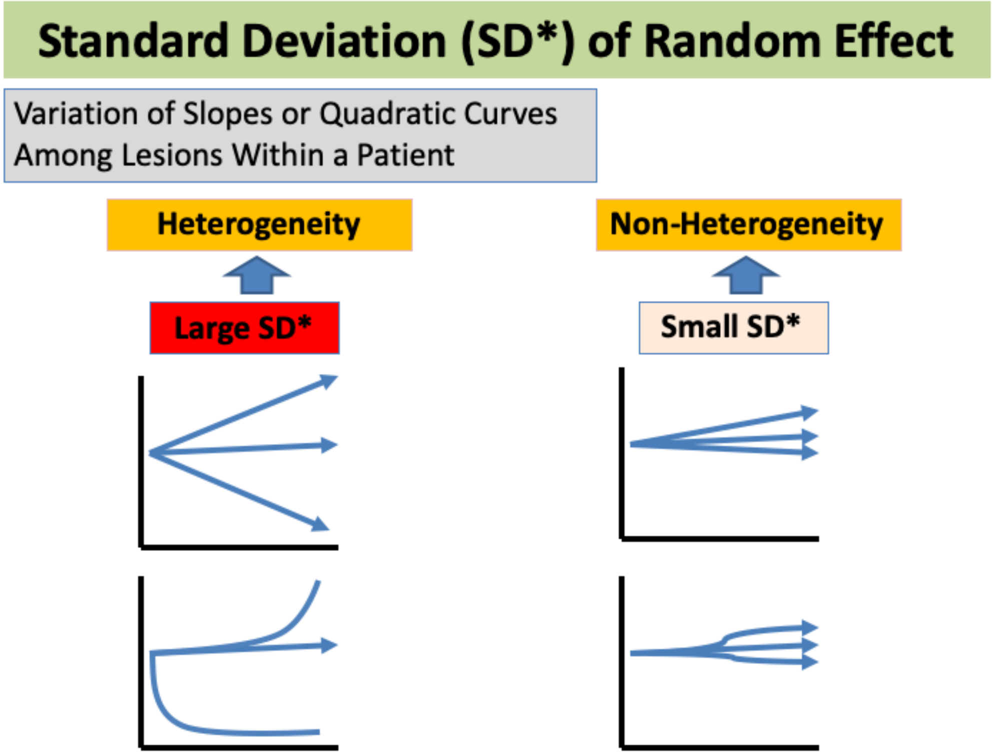

SD*: Since frequency of tumor measurement (CT scan frequency) is different in each patient, statistical model is adaptively changed according to the measurement frequency. For cases with three CT scans, lesion profile is analyzed by a linear slope regression in a mixed effect model. Standard deviation (SD*) of the random component in the linear slope is used to assess lesion variation. For patients with 4 CT scans or more, a mixed effect model using a quadratic function is applied for analysis. Similarly, the derived SD*s from the random components in both linear slope and quadratic term are used to determine lesion heterogeneity. If a patient has the largest estimated SD*s above a threshold, δ, lesion heterogeneity will be claimed.

S2 Fig. Modified RECIST response

Figure S2: Modified RECIST response

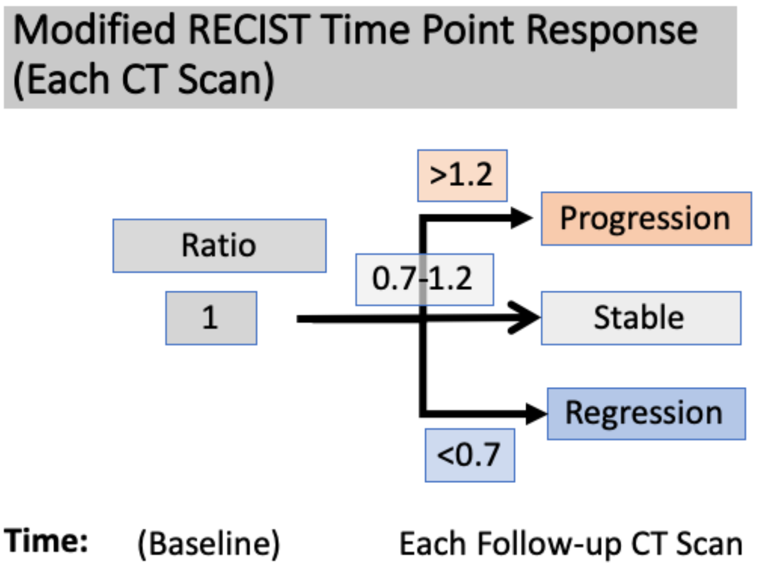

S3 Fig. Mean of square deviations (MSD) to assess classification performance

Figure S3:

(A) Definition of various MSDs

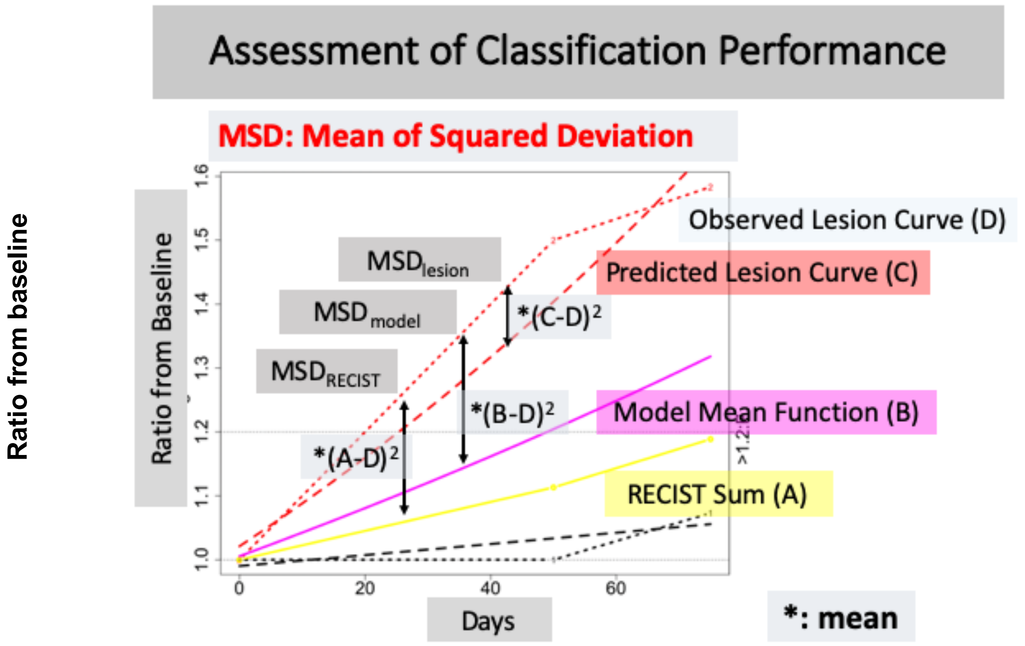

(B) MSD as a metric to assess classification performance

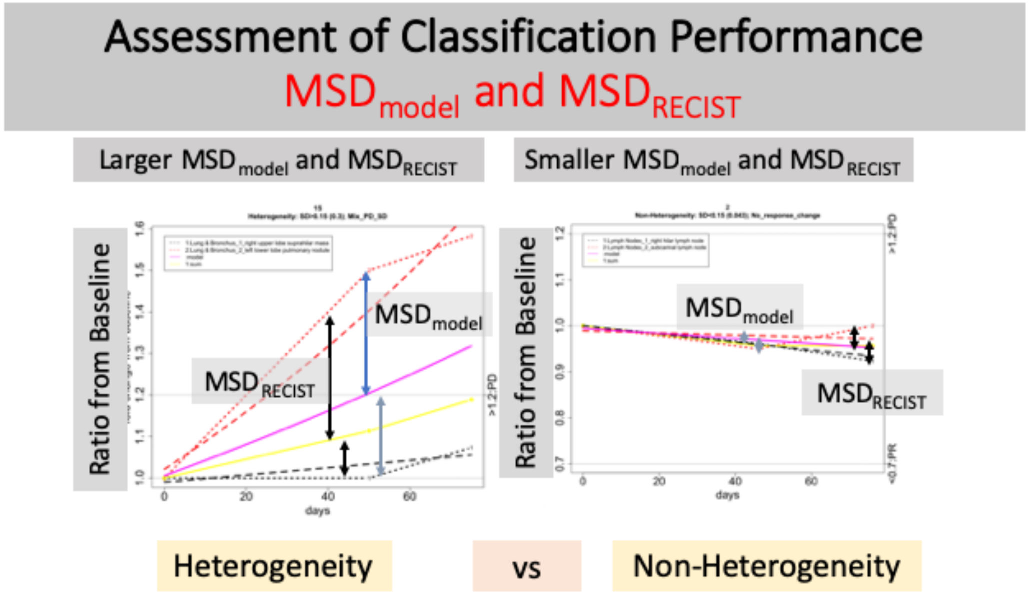

S4 Fig. Detection of outlier lesion

Figure S4: Detection of outlier lesion

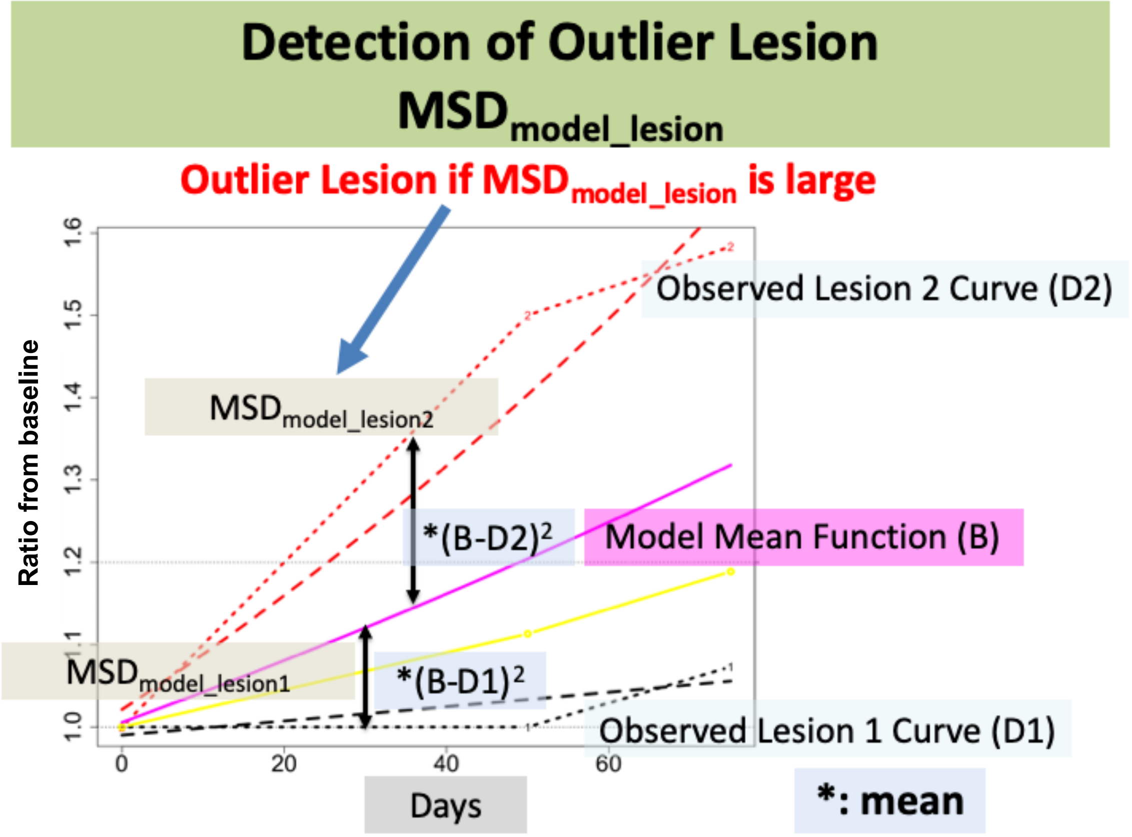

S5 Fig. Model goodness of fit

Figure S5: Model goodness of fit

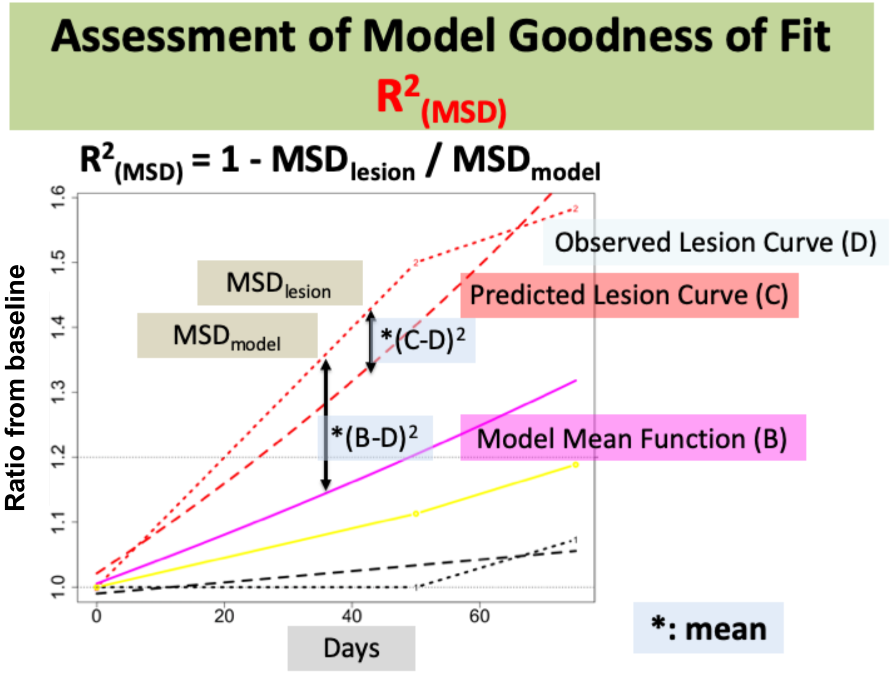

R^2_{MSD} is used to assess model goodness of fit for the heterogeneity group and defined as $R^2_{MSD} = 1 - {}^{MSD_{lesion}}\!/_{MSD_{model}}$. It measures how much deviation of all predicted lesion curves away from the observed lesion curves in a patient (*MSD*$_{(lesion)}$). A value of *MSD*$_{(lesion)}$ close to 0 indicates good fit of data while a large value implies poor prediction. Specifically, R^2_{MSD} evaluates deviation of the model mean function from the observed lesion curves (*MSD*$_{(model)}$) which is used as the reference to adjust for *MSD*$_{(lesion)}$ due to unique lesion growth profiles in each patient. Adjustment is made by taking a ratio, *MSD*$_{(lesion)}$ /*MSD*$_{(model)}$. The *MSD*$_{(model)}$ is expected large in the lesion heterogeneity group because of diverse tumor growth patterns among lesions. When *MSD*$_{(lesion)}$ value is close to *MSD*$_{(model)}$ value, the ratio is close to 1 indicating the predicted lesion curves do not model well the observed lesion curves. So R^2_{MSD} will be near to 0. On the other hand, if *MSD*$_{(lesion)}$ value is different from *MSD*$_{(model)}$ value and close to 0, the ratio will approach 0 and R^2_{MSD} will be close to 1, suggesting a good fit of the predicted lesion curves.

S6 Fig.

(S6A): Justification of the SD* threshold

Figure S6A: Justification of the SD* threshold

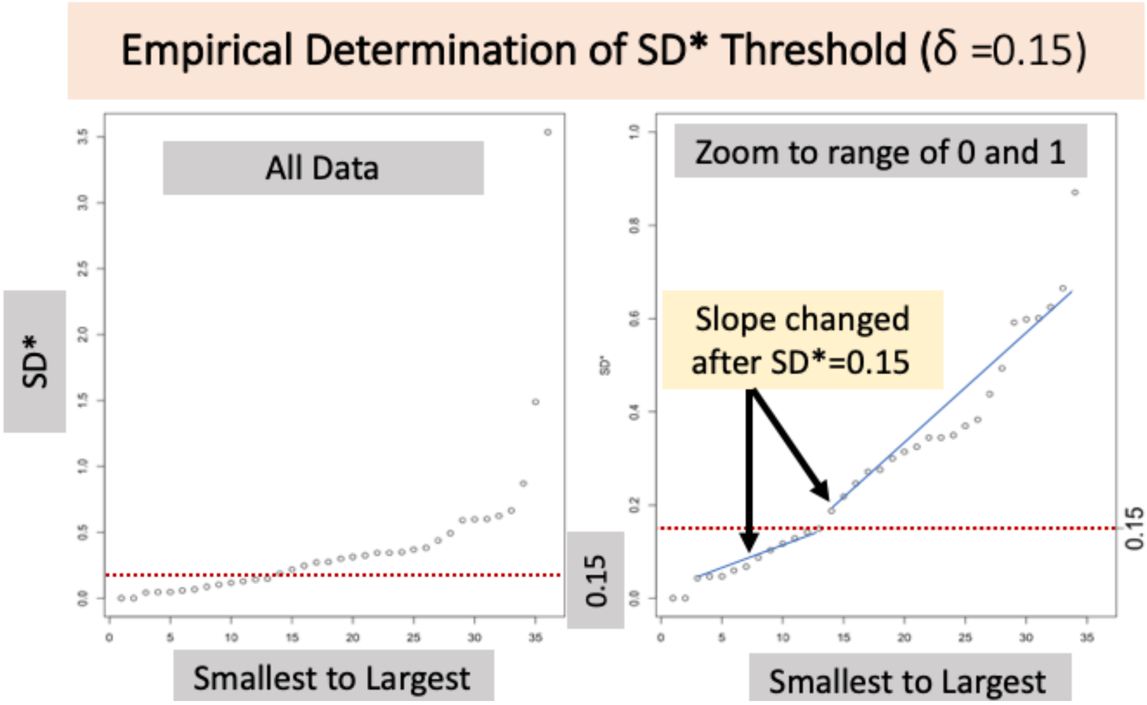

(S6B): Comparable performance of $MSD_{(RECIST)}$ and $MSD_{(model)}$

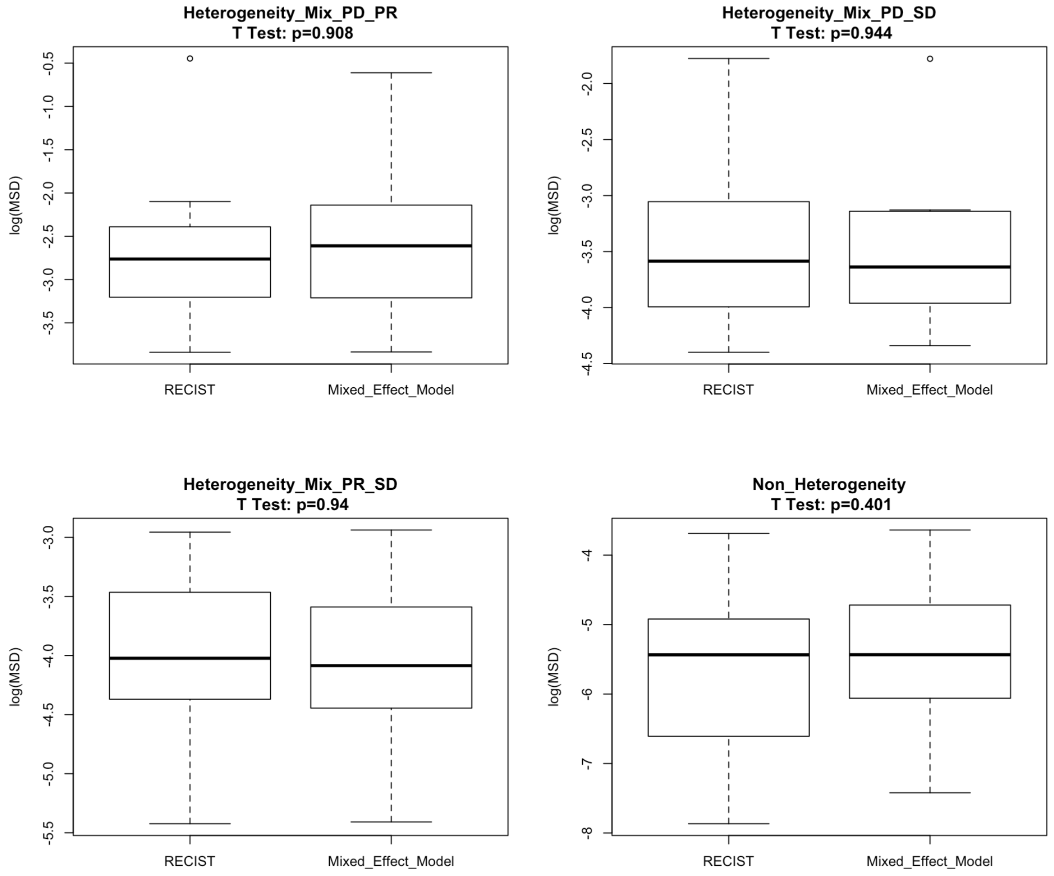

Figure S6B: Comparable performance of $MSD_{(RECIST)}$ and $MSD_{(model)}$

Mix_PD_PR: Mix of progressive and regressive lesions
Mix_PD_SD: Mix of progressive and stable lesions
Mix_PR_SD: Mix of regressive and stable lesions

S7 Fig. Frequency of outlier lesions among heterogenous subgroups

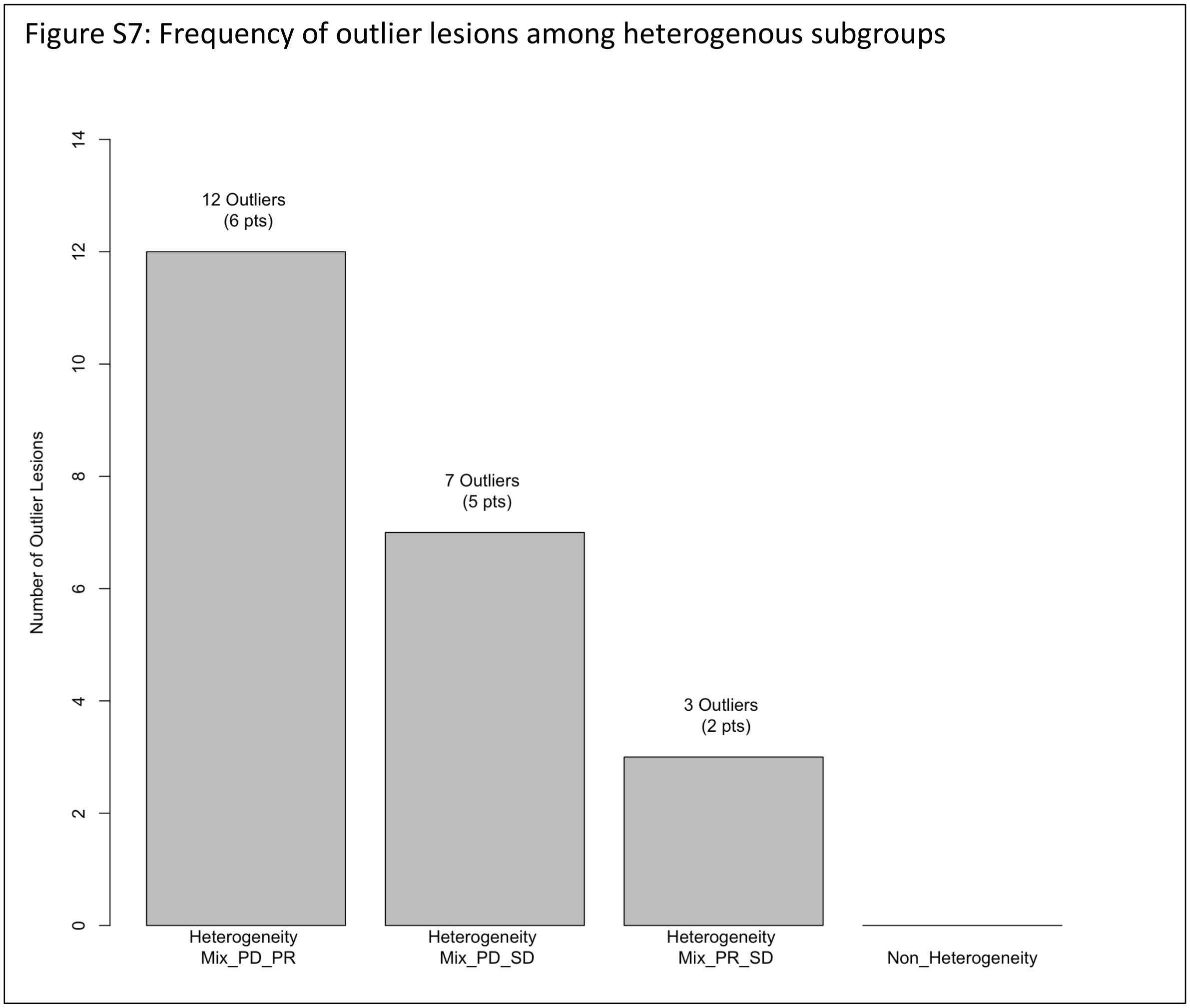

Mix_PD_PR: Mix of progressive and regressive lesions
Mix_PD_SD: Mix of progressive and stable lesions
Mix_PR_SD: Mix of regressive and stable lesions

S8 Fig. Evaluation of model goodness of fit

Figure S8: Evaluation of model goodness of fit

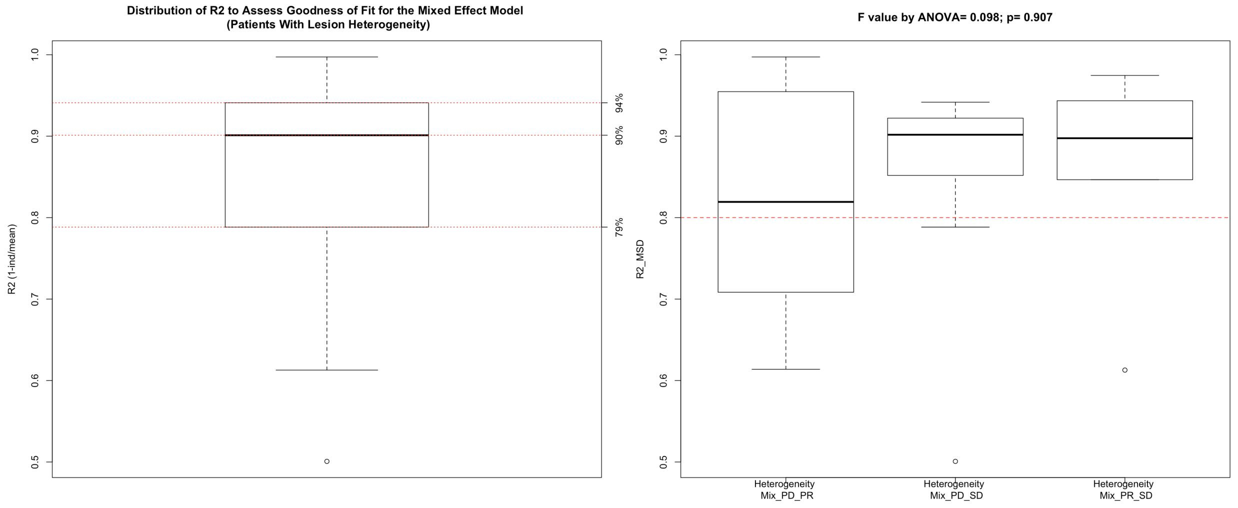

Mix_PD_PR: Mix of progressive and regressive lesions
Mix_PD_SD: Mix of progressive and stable lesions
Mix_PR_SD: Mix of regressive and stable lesions

S9 Fig. Sporadic mixed response

Figure S9: Sporadic mixed response

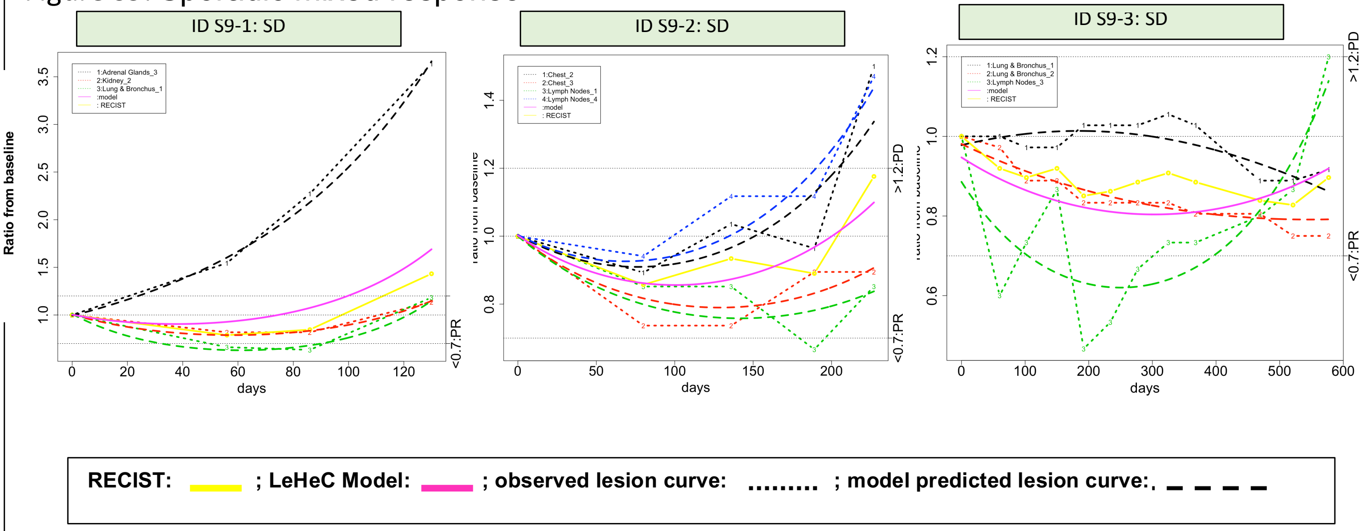

The sporadic mixed response had some progressive lesions and some stable lesions with temporary regression at some time points (ID S9-1 and ID S9-2). It could also be some lesions experienced regression to progression over time (ID S9-3).

S10 Fig.

(S10A): Mix of progressive and stable lesions (Most stable lesions with a few progressive lesions, oligometastasis-like pattern)

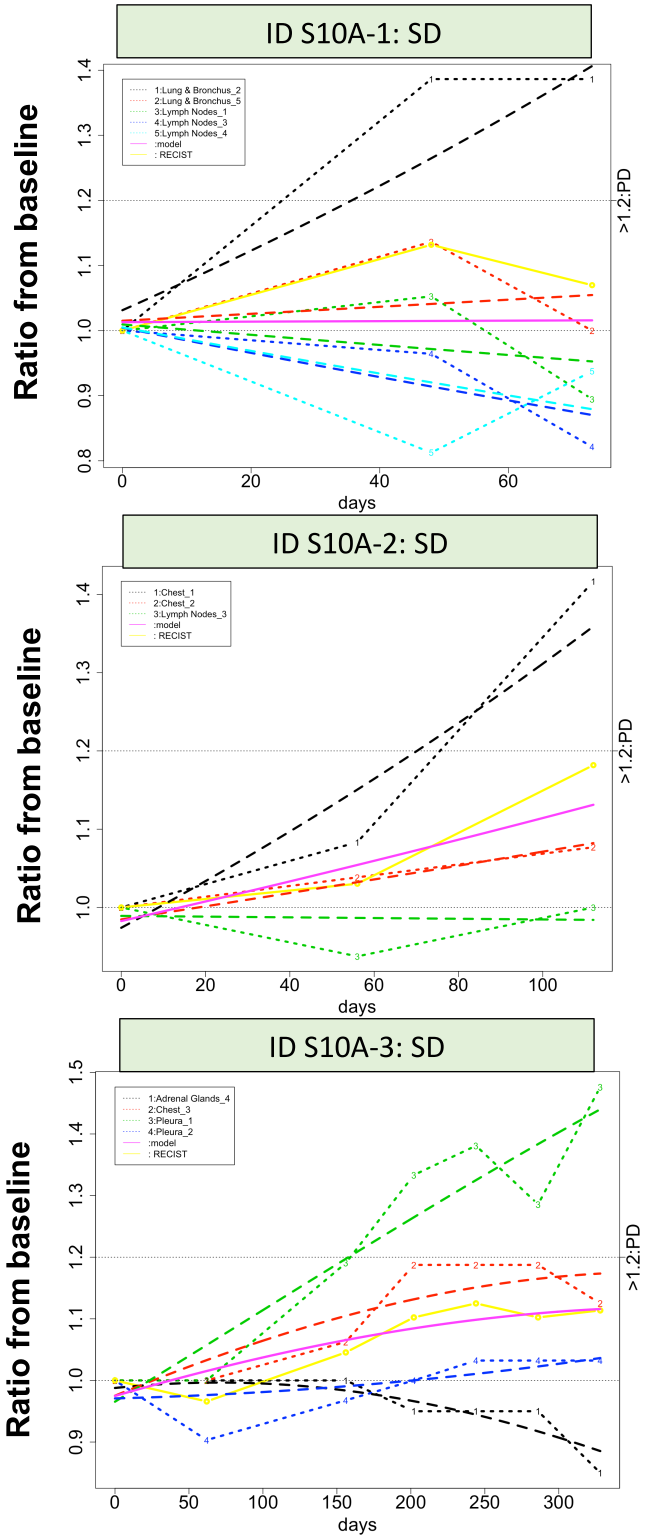

RECIST sum: ; LeHeC Model: ; observed lesion curve: ; model predicted lesion curve:

(S10B): Mix of progressive and stable lesions (Other patterns)

(i) Most progressive lesions and few stable lesions

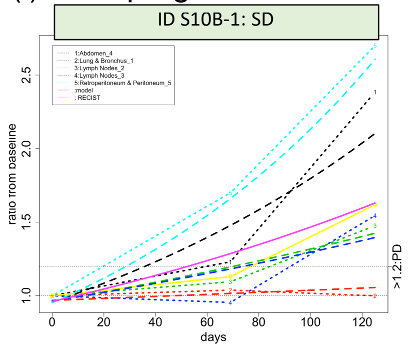

(ii) Balanced mix of progression and stable lesions

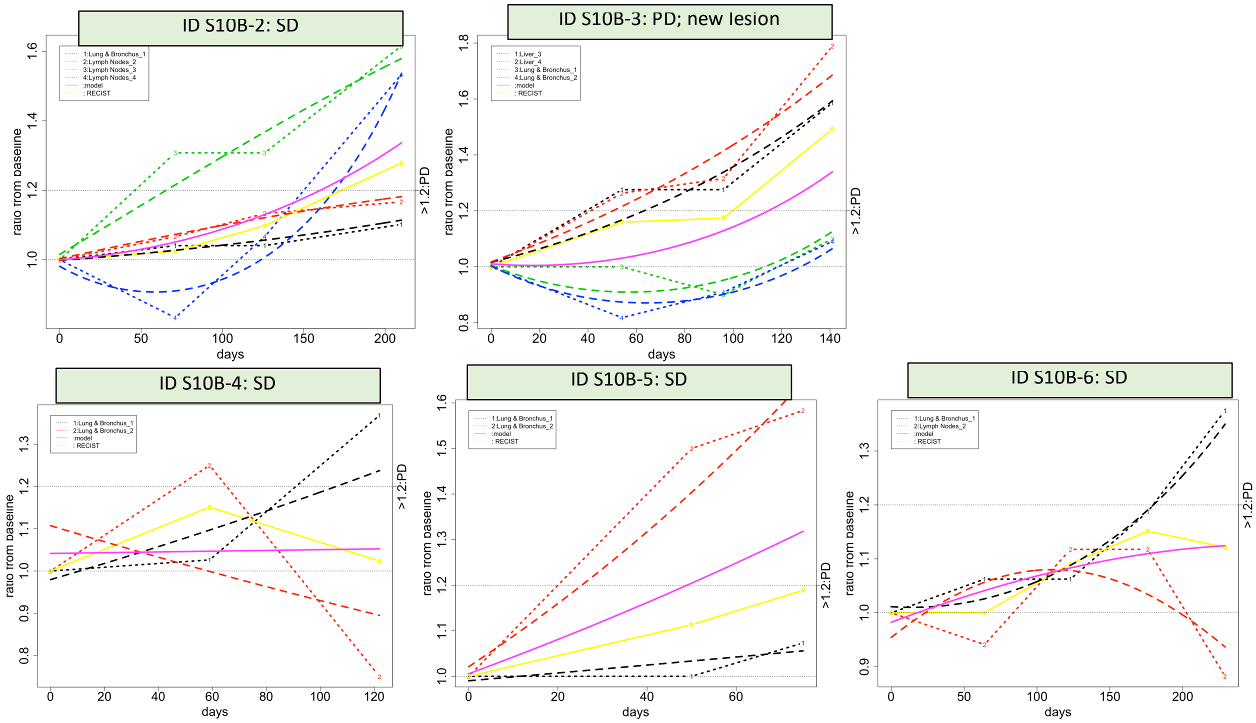

RECIST sum: ; **LeHeC Model:** ; **observed lesion curve:** .... ; **model predicted lesion curve:** — — — —

One pattern had an opposite case with most progressive lesions and few stable lesions (ID S10B-1). The other pattern was a balanced mix of progression and stable lesions (ID S10B-2, ID S10B-3, ID S10B-4, ID S10B-5, ID S10B-6).

S11 Fig.
(S11A): Mix of regressive and stable lesions

Figure S11A: Mix of regressive and stable lesions

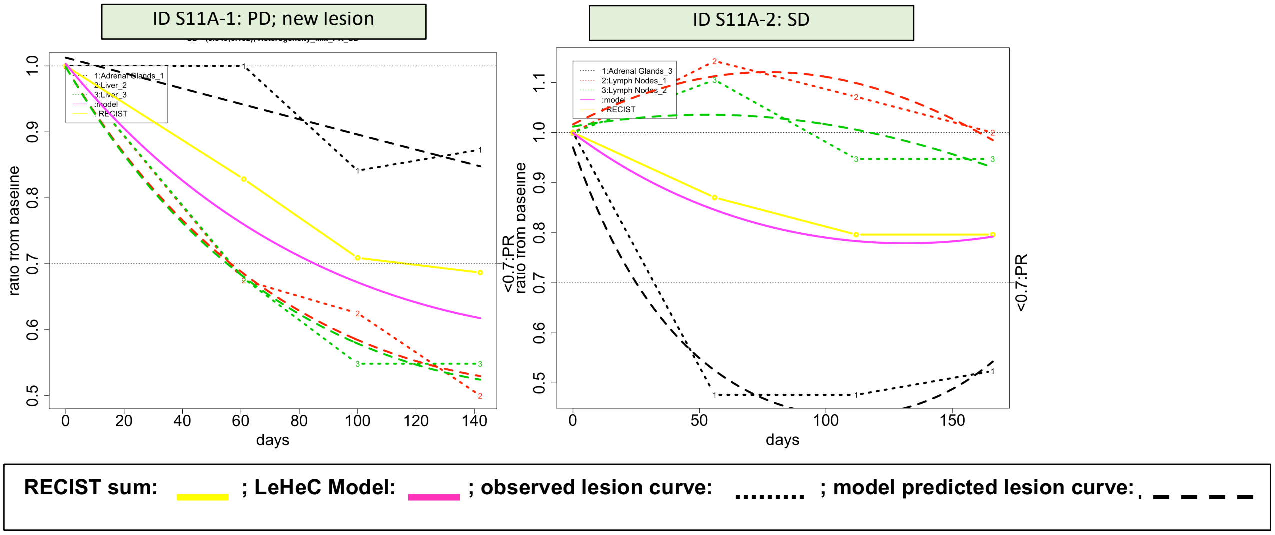

The classic pattern was that some lesions had tumor reduction while others remained unchanged (regular pattern).

(S11B): Mix of regressive and stable lesions

Figure S11B: Mix of regressive and stable lesions

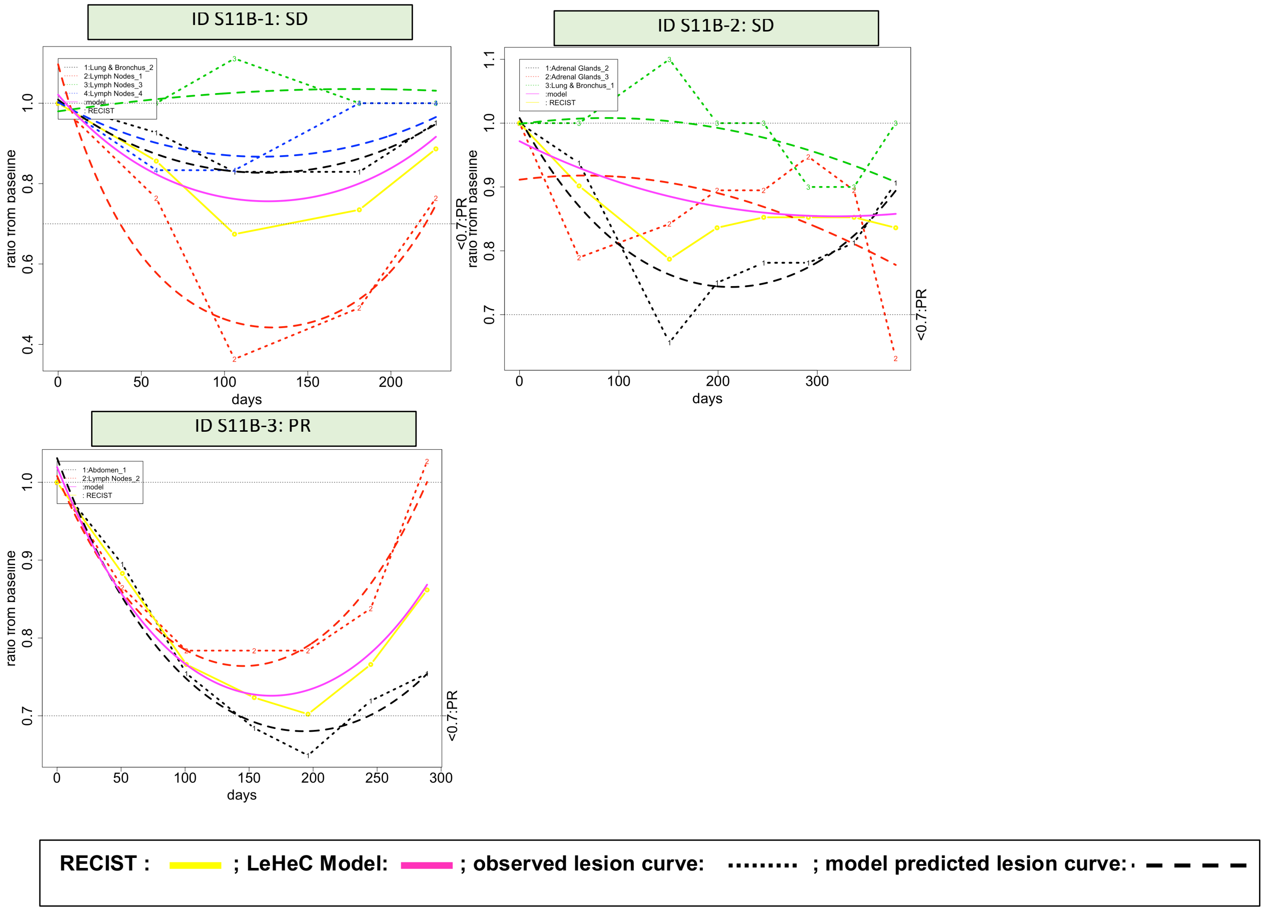

The other pattern was that some lesions experienced tumor reduction at least 30% in some time points while other lesions were relatively unchanged with a ratio between 0.7 and 1.2.

S12 Fig.
(S12A): Non-Heterogeneity (Stable)

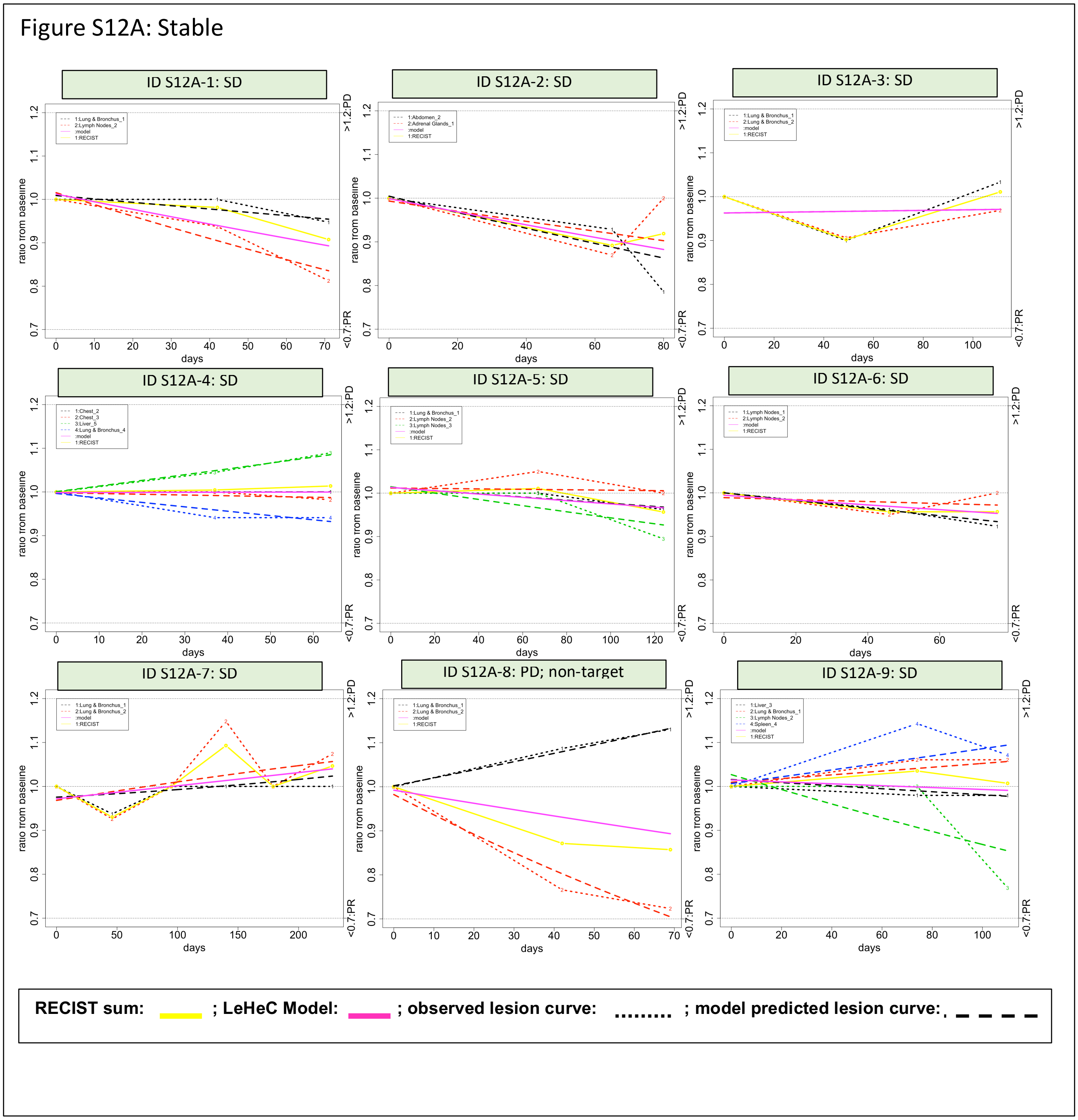

(S12B): Non-Heterogeneity (Similar growth trend)

Figure S12B: Similar growth trend

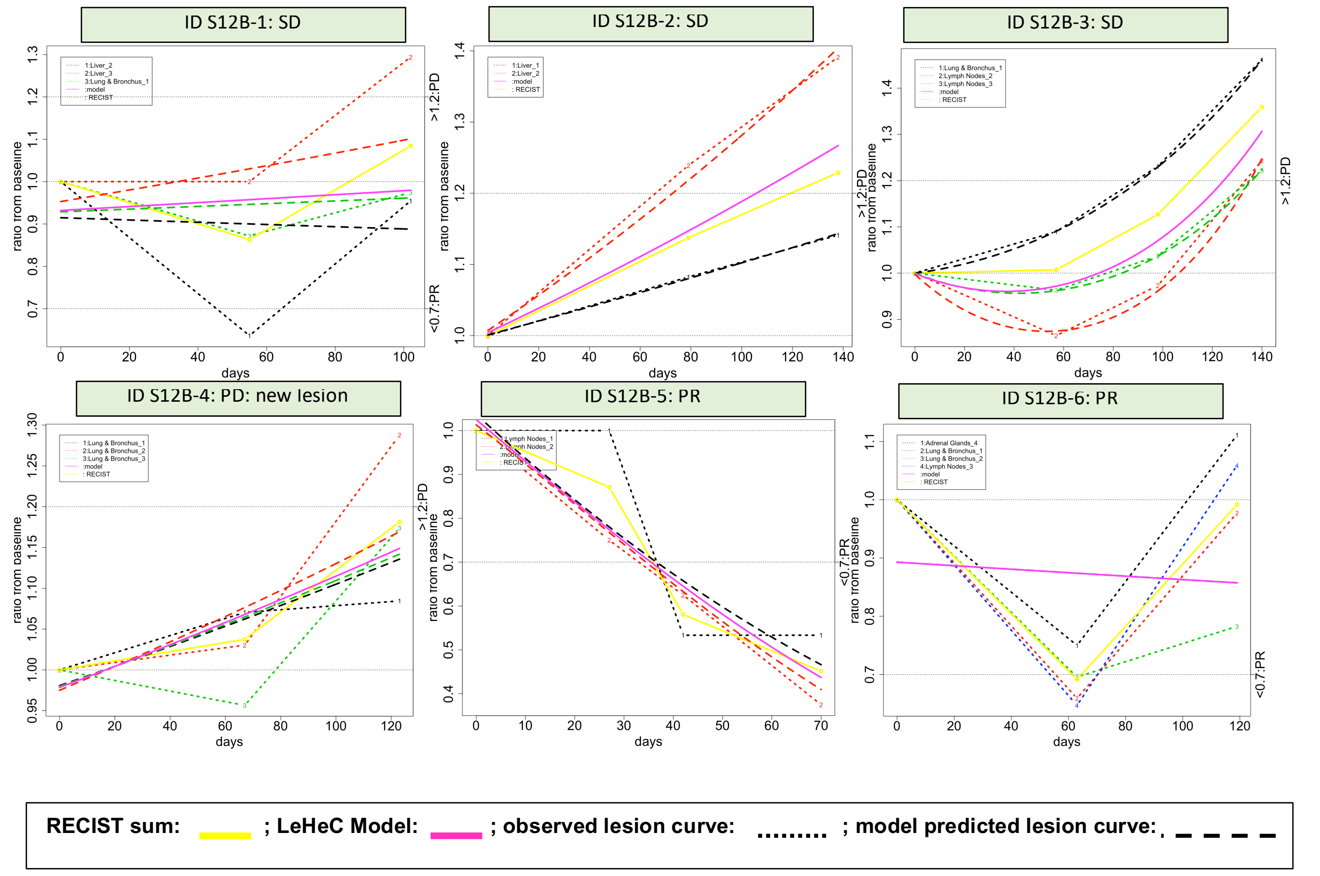

R code for lesion heterogeneity

```
lesion.heterogeneity.fun<-
function(data.tmp.list,tiff.status=F,plot.staus=T,plot.folder=NULL,plot.prediction=T,legend.x=0,l
wd.tmp=4,cex.main.tmp=3,time.unit='day',truncated.time=NULL)
{
  require(tidyverse)
  require(nlme)
  require(lme4)

    coef.tmp<-numeric(0)
  lmer.est.list<-time.response.list<-rss.mean.lesion<-list()
  rss.mean.tmp<-numeric()
  for(i in 1:length(data.tmp.list))
  {
    tmp1<-data.tmp.list[[i]]$lesion
    f1.time<-tmp1$time
    y.min<-10^(-2)
    tmp1$tumor_ratio.org<-tmp1$tumor_ratio
    tmp1$tumor_ratio<-ifelse(tmp1$tumor_ratio==0,log(y.min),log(tmp1$tumor_ratio))
    f1.y<-tmp1$tumor_ratio
    tmp.wide.ratio<-tmp1%>%select(site,time,tumor_ratio)%>%spread(site,tumor_ratio,drop=T)
    f1.time.sum<-tmp.wide.ratio[,1]
    tmp.wide.ratio<-tmp.wide.ratio[,-1]
    sum.tmp<-data.tmp.list[[i]]$RECIST.sum$tumor_ratio
    sum.tmp<-ifelse(sum.tmp==0,log(y.min),log(sum.tmp))
    rss.sum<-as.matrix((exp(tmp.wide.ratio)-exp(sum.tmp)))

    tt0<-seq(min(f1.time.sum),max(f1.time.sum),by=1)
    time.response.tmp<-apply(tmp.wide.ratio,2,function(x) cut(x,breaks = log(c(10^(-10),.7,1.2-
10^(-10),100)),label=c('PR','SD','PD')))
    time.response.list[[i]]<-time.response.tmp
    tmp.wide<-tmp.wide.ratio

    tmp1.new <-groupedData( tumor_ratio ~ time | site,data = tmp1)
    tmp1.new$site<-factor(as.vector(tmp1.new$site))

    #---linear mixed effect---
    if(length(table(tmp1.new$time))>3)
    {
      lmer.est<-lmer(tumor_ratio ~ poly(time,2)+(poly(time,2)|site),data = tmp1.new)
    }
    if(length(table(tmp1.new$time))==3)
```

```
    {
      lmer.est<-lmer(tumor_ratio ~ poly(time,1)+(poly(time,1)|site),data = tmp1.new)
    }
    lmer.predict.tmp<-apply(as.matrix(names(table(tmp1.new$site))),1,function(x)
{data2=data.frame(site=x,time=tt0);predict(lmer.est,data2)})
    lmer.predict.ind.tmp<-apply(as.matrix(names(table(tmp1.new$site))),1,function(x)
{data2=data.frame(site=x,time=f1.time.sum);predict(lmer.est,data2)})
    lmer.predict.mean.tmp=predict(lmer.est,data.frame(time=f1.time.sum),re.form=~0)

    random.sd<-attr(summary(lmer.est)$varcor$site,"stddev")[-1]

    index1<-apply(time.response.tmp,1,function(y)length(table(y)))
    PD.any<-apply(time.response.tmp,1,function(x) any(x=='PD'))
    PRCR.any<-apply(time.response.tmp,1,function(x) any(x=='PR'|x=='CR'))
    SD.all<-apply(time.response.tmp,1,function(x) all(x=='SD'))

    if(any(index1>1))
    {
      if(any(PD.any)&any(PRCR.any)) response.tmp<-'Heterogeneity_Mix_PD_PR' else
      {
        if(any(PD.any)) response.tmp<-'Heterogeneity_Mix_PD_SD'
        if(any(PRCR.any)) response.tmp<-'Heterogeneity_Mix_PR_SD'
      }

    } else  response.tmp<-'Non_Heterogeneity_Stable'

    if(!any(random.sd>0.15)) response.tmp<-
paste('Non_Heterogeneity',ifelse(all(SD.all),'Stable','Similar Growth Trend'),sep='_')
    group.response.tmp<-response.tmp

    rss.lmer.mean<-as.matrix((exp(tmp.wide.ratio)-exp(lmer.predict.mean.tmp)))
    rss.lmer.ind<-as.matrix((exp(tmp.wide.ratio)-exp(lmer.predict.ind.tmp)))

    rss.mean.tmp.ind<-
data.frame(id=as.vector(tmp1$id)[1],random.sd=max(random.sd),group=group.response.tmp,
                         rss.sum.mean.with_baseline=mean(rss.sum^2),
                         rss.sum.mean.no_baseline=mean(rss.sum[-1,]^2),

                         rss.lmer.mean.with_baseline=mean(rss.lmer.mean^2),
                         rss.lmer.mean.no_baseline=mean(rss.lmer.mean[-1,]^2),

                         rss.lmer.ind.with_baseline=mean(rss.lmer.ind^2),
                         rss.lmer.ind.no_baseline=mean(rss.lmer.ind[-1,]^2)
    )
```

```
    rss.mean.tmp<-rbind(rss.mean.tmp,rss.mean.tmp.ind)

    rss.mean.lesion[[i]]<-data.frame(id=as.vector(tmp1$id)[1],
                                     rss.sum.mean.lesion=apply(rss.sum^2,2,mean),
                                     rss.lmer.mean.lesion=apply(rss.lmer.mean^2,2,mean),
                                     rss.lmer.ind.lesion=apply(rss.lmer.ind^2,2,mean)
    )

    lmer.est.list[[i]]<-lmer.est

    if(tiff.status & (!is.null(plot.folder)))
    {
      dir0<-plot.folder
      if(!dir.exists(dir0)) dir.create(dir0)
      plot.dir.tmp<-paste(dir0,as.vector(rss.mean.tmp.ind$group[1]),'/',sep='')
      if(!dir.exists(plot.dir.tmp)) dir.create(plot.dir.tmp)
      png(filename =
paste(plot.dir.tmp,'sd_',sub('\\.','_',round(rss.mean.tmp.ind$random.sd[1],3)),'_',rss.mean.tmp.
ind$id[1],'.png',sep=''),width = 1920, height = 1680,res=180)
    }


    if(plot.staus)
    {
        fold.tmp<-exp(tmp.wide.ratio)
        nn.tmp<-dim(fold.tmp)[2]

        xlab.tmp<-'days'
        truncated.time.tmp<-ifelse(is.null(truncated.time),max(f1.time.sum),truncated.time)

        if(time.unit=='month')
          {
            f1.time.sum<-f1.time.sum/30
            xlab.tmp<-'months'
            truncated.time.tmp<-ifelse(is.null(truncated.time),max(f1.time.sum),truncated.time)
        }
        xlim.tmp<-c(0,truncated.time.tmp)

        if((min(fold.tmp)>0.7)&(max(fold.tmp)<1.2))
        {
          matplot(f1.time.sum,fold.tmp,type='b',xlab=xlab.tmp,ylab='ratio from
baseline',lwd=lwd.tmp,lty=3,cex.lab=2,cex.axis=2,ylim=c(0.7,1.2),xlim=xlim.tmp)
```

```
legend(legend.x,1.2,paste(c(1:nn.tmp,''),':',c(colnames(tmp.wide.ratio),'model','RECIST'),sep=''),
col=c(1:nn.tmp,6,7),lty=c(rep(2,nn.tmp),c(1,1)))}
       else
       {
         matplot(f1.time.sum,fold.tmp,type='b',xlab=xlab.tmp,ylab='ratio from
baseline',lwd=lwd.tmp,lty=3,cex.lab=2,cex.axis=2,xlim=xlim.tmp)
         if(plot.prediction)
legend(legend.x,max(exp(tmp.wide.ratio)),paste(c(1:nn.tmp,'',''),':',c(colnames(tmp.wide.ratio),
'model',' RECIST'),sep=''),col=c(1:nn.tmp,6,7),lty=c(rep(3,nn.tmp),c(1,1))) else

legend(legend.x,max(exp(tmp.wide.ratio)),paste(c(1:nn.tmp,''),':',c(colnames(tmp.wide.ratio),'
RECIST'),sep=''),col=c(1:nn.tmp,7),lty=c(rep(3,nn.tmp),c(1)))
       }

      lines(f1.time.sum,exp(sum.tmp),lwd=lwd.tmp,col=7,type='b')

      abline(h=c(0.7,1,1.2),lty=3)
      axis(4,c(0.7,1.2),label=c('<0.7:PR','>1.2:PD'),cex.axis=2)
      if(plot.prediction) matlines(tt0,exp(lmer.predict.tmp),lwd=lwd.tmp,lty=2)
      data2=data.frame(time=tt0)
      # this for lme4: lmer
      r1=predict(lmer.est,data2,re.form=~0)
      if(plot.prediction) lines(tt0,exp(r1),col=6,lty=1,lwd=lwd.tmp)

      if(plot.prediction)
title(paste(tmp1.new$id[1],'\n','SD*=(',paste(round(random.sd,3),collapse=';'),");
",group.response.tmp,sep=''),cex.main=cex.main.tmp) else
      title(group.response.tmp,cex.main=cex.main.tmp)

    }
    if(tiff.status)    dev.off()
  }
  names(lmer.est.list)<-names(time.response.list)<-names(rss.mean.lesion)<-
names(data.tmp.list)


list(lmer.est.list=lmer.est.list,time.response.list=time.response.list,rss.mean.lesion=rss.mean.les
ion,data.tmp.list=data.tmp.list,rss.mean.tmp=rss.mean.tmp)
}

data2<-read.csv('Data/data_manscript.csv')
data2.sum<-read.csv('Data/data_RECIST_sum_manscript.csv',skip=1)
data30=by(data2,data2$id,data.frame)
```

```
data31<-by(data2.sum,data2.sum$id,data.frame)
data3<-list()
for(i in 1:length(data30))
{
  data3[[i]]<-list()
  data3[[i]]$lesion<-data30[[i]]
  data3[[i]]$RECIST.sum<-data31[[i]]
}
names(data3)<-names(data30)

ans=lesion.heterogeneity.fun(data.tmp.list = data3,tiff.status = T,cex.main.tmp=1,plot.folder =
'plot/png_manuscript_test1/')

#---for psuedo-progression: ID3 and ID4---
ans1=lesion.heterogeneity.fun(data.tmp.list = data3[c(35,36)],tiff.status = T,plot.folder =
'plot/png_manuscript_new/',legend.x=100)

#---no prediction line----
ans=lesion.heterogeneity.fun(data.tmp.list = data3,tiff.status = T,plot.prediction = F,lwd.tmp=8,
time.unit='month',truncated.time=NULL,plot.folder = 'plot/png_no_prediciton/')
#---for psuedo-progression: ID3 and ID4---
ans1=lesion.heterogeneity.fun(data.tmp.list = data3[c(35,36)],tiff.status = T,plot.prediction =
F,lwd.tmp = 8, time.unit='month',truncated.time=NULL,plot.folder =
'plot/png_no_prediciton/',legend.x=100)
```

Lesion level data

id	site	time	tumor_ratio
ID S10A-1	Lung & Bronchus_2	0	1
ID S10A-1	Lung & Bronchus_2	48	1.38636364
ID S10A-1	Lung & Bronchus_2	73	1.38636364
ID S10A-1	Lung & Bronchus_5	0	1
ID S10A-1	Lung & Bronchus_5	48	1.13636364
ID S10A-1	Lung & Bronchus_5	73	1
ID S10A-1	Lymph Nodes_1	0	1
ID S10A-1	Lymph Nodes_1	48	1.05263158
ID S10A-1	Lymph Nodes_1	73	0.89473684
ID S10A-1	Lymph Nodes_3	0	1
ID S10A-1	Lymph Nodes_3	48	0.96428571
ID S10A-1	Lymph Nodes_3	73	0.82142857
ID S10A-1	Lymph Nodes_4	0	1
ID S10A-1	Lymph Nodes_4	48	0.8125
ID S10A-1	Lymph Nodes_4	73	0.9375
ID S10A-2	Chest_1	0	1
ID S10A-2	Chest_1	56	1.08333333
ID S10A-2	Chest_1	112	1.41666667
ID S10A-2	Chest_2	0	1
ID S10A-2	Chest_2	56	1.03846154
ID S10A-2	Chest_2	112	1.07692308
ID S10A-2	Lymph Nodes_3	0	1
ID S10A-2	Lymph Nodes_3	56	0.9375
ID S10A-2	Lymph Nodes_3	112	1
ID S10A-3	Adrenal Glands_4	0	1
ID S10A-3	Adrenal Glands_4	62	1
ID S10A-3	Adrenal Glands_4	156	1
ID S10A-3	Adrenal Glands_4	202	0.95
ID S10A-3	Adrenal Glands_4	244	0.95
ID S10A-3	Adrenal Glands_4	286	0.95
ID S10A-3	Adrenal Glands_4	328	0.85
ID S10A-3	Chest_3	0	1
ID S10A-3	Chest_3	62	1
ID S10A-3	Chest_3	156	1.0625
ID S10A-3	Chest_3	202	1.1875
ID S10A-3	Chest_3	244	1.1875
ID S10A-3	Chest_3	286	1.1875

ID S10A-3	Chest_3	328	1.125
ID S10A-3	Pleura_1	0	1
ID S10A-3	Pleura_1	62	1
ID S10A-3	Pleura_1	156	1.19047619
ID S10A-3	Pleura_1	202	1.33333333
ID S10A-3	Pleura_1	244	1.38095238
ID S10A-3	Pleura_1	286	1.28571429
ID S10A-3	Pleura_1	328	1.47619048
ID S10A-3	Pleura_2	0	1
ID S10A-3	Pleura_2	62	0.90322581
ID S10A-3	Pleura_2	156	0.96774194
ID S10A-3	Pleura_2	202	1
ID S10A-3	Pleura_2	244	1.03225806
ID S10A-3	Pleura_2	286	1.03225806
ID S10A-3	Pleura_2	328	1.03225806
ID S10B-1	Abdomen_4	0	1
ID S10B-1	Abdomen_4	69	1.23076923
ID S10B-1	Abdomen_4	125	2.38461538
ID S10B-1	Lung & Bronchus_1	0	1
ID S10B-1	Lung & Bronchus_1	69	1.03846154
ID S10B-1	Lung & Bronchus_1	125	1
ID S10B-1	Lymph Nodes_2	0	1
ID S10B-1	Lymph Nodes_2	69	1.0952381
ID S10B-1	Lymph Nodes_2	125	1.47619048
ID S10B-1	Lymph Nodes_3	0	1
ID S10B-1	Lymph Nodes_3	69	0.95454545
ID S10B-1	Lymph Nodes_3	125	1.54545455
ID S10B-1	Retroperitoneum & Peritoneum_5	0	1
ID S10B-1	Retroperitoneum & Peritoneum_5	69	1.7
ID S10B-1	Retroperitoneum & Peritoneum_5	125	2.7
ID S10B-2	Lung & Bronchus_1	0	1
ID S10B-2	Lung & Bronchus_1	71	1.04081633
ID S10B-2	Lung & Bronchus_1	126	1.04081633
ID S10B-2	Lung & Bronchus_1	210	1.10204082
ID S10B-2	Lymph Nodes_2	0	1
ID S10B-2	Lymph Nodes_2	71	1.06666667
ID S10B-2	Lymph Nodes_2	126	1.13333333
ID S10B-2	Lymph Nodes_2	210	1.16666667

ID S10B-2	Lymph Nodes_3	0	1
ID S10B-2	Lymph Nodes_3	71	1.30769231
ID S10B-2	Lymph Nodes_3	126	1.30769231
ID S10B-2	Lymph Nodes_3	210	1.61538462
ID S10B-2	Lymph Nodes_4	0	1
ID S10B-2	Lymph Nodes_4	71	0.83333333
ID S10B-2	Lymph Nodes_4	126	1.06666667
ID S10B-2	Lymph Nodes_4	210	1.53333333
ID S10B-3	Liver_3	0	1
ID S10B-3	Liver_3	54	1.27586207
ID S10B-3	Liver_3	96	1.27586207
ID S10B-3	Liver_3	141	1.5862069
ID S10B-3	Liver_4	0	1
ID S10B-3	Liver_4	54	1.26315789
ID S10B-3	Liver_4	96	1.31578947
ID S10B-3	Liver_4	141	1.78947368
ID S10B-3	Lung & Bronchus_1	0	1
ID S10B-3	Lung & Bronchus_1	54	1
ID S10B-3	Lung & Bronchus_1	96	0.9
ID S10B-3	Lung & Bronchus_1	141	1.1
ID S10B-3	Lung & Bronchus_2	0	1
ID S10B-3	Lung & Bronchus_2	54	0.81818182
ID S10B-3	Lung & Bronchus_2	96	0.90909091
ID S10B-3	Lung & Bronchus_2	141	1.09090909
ID S10B-4	Lung & Bronchus_1	0	1
ID S10B-4	Lung & Bronchus_1	59	1.02631579
ID S10B-4	Lung & Bronchus_1	122	1.36842105
ID S10B-4	Lung & Bronchus_2	0	1
ID S10B-4	Lung & Bronchus_2	59	1.25
ID S10B-4	Lung & Bronchus_2	122	0.75
ID S10B-5	Lung & Bronchus_1	0	1
ID S10B-5	Lung & Bronchus_1	50	1
ID S10B-5	Lung & Bronchus_1	75	1.07317073
ID S10B-5	Lung & Bronchus_2	0	1
ID S10B-5	Lung & Bronchus_2	50	1.5
ID S10B-5	Lung & Bronchus_2	75	1.58333333
ID S10B-6	Lung & Bronchus_1	0	1
ID S10B-6	Lung & Bronchus_1	64	1.0625
ID S10B-6	Lung & Bronchus_1	123	1.0625
ID S10B-6	Lung & Bronchus_1	176	1.1875

ID S10B-6	Lung & Bronchus_1	229	1.375
ID S10B-6	Lymph Nodes_2	0	1
ID S10B-6	Lymph Nodes_2	64	0.94117647
ID S10B-6	Lymph Nodes_2	123	1.11764706
ID S10B-6	Lymph Nodes_2	176	1.11764706
ID S10B-6	Lymph Nodes_2	229	0.88235294
ID S11A-1	Adrenal Glands_1	0	1
ID S11A-1	Adrenal Glands_1	61	1
ID S11A-1	Adrenal Glands_1	100	0.84126984
ID S11A-1	Adrenal Glands_1	142	0.87301587
ID S11A-1	Liver_2	0	1
ID S11A-1	Liver_2	61	0.675
ID S11A-1	Liver_2	100	0.625
ID S11A-1	Liver_2	142	0.5
ID S11A-1	Liver_3	0	1
ID S11A-1	Liver_3	61	0.67741935
ID S11A-1	Liver_3	100	0.5483871
ID S11A-1	Liver_3	142	0.5483871
ID S11A-2	Adrenal Glands_3	0	1
ID S11A-2	Adrenal Glands_3	56	0.47619048
ID S11A-2	Adrenal Glands_3	112	0.47619048
ID S11A-2	Adrenal Glands_3	166	0.52380952
ID S11A-2	Lymph Nodes_1	0	1
ID S11A-2	Lymph Nodes_1	56	1.14285714
ID S11A-2	Lymph Nodes_1	112	1.07142857
ID S11A-2	Lymph Nodes_1	166	1
ID S11A-2	Lymph Nodes_2	0	1
ID S11A-2	Lymph Nodes_2	56	1.10526316
ID S11A-2	Lymph Nodes_2	112	0.94736842
ID S11A-2	Lymph Nodes_2	166	0.94736842
ID S11B-1	Lung & Bronchus_2	0	1
ID S11B-1	Lung & Bronchus_2	59	0.92682927
ID S11B-1	Lung & Bronchus_2	106	0.82926829
ID S11B-1	Lung & Bronchus_2	181	0.82926829
ID S11B-1	Lung & Bronchus_2	227	0.95121951
ID S11B-1	Lymph Nodes_1	0	1
ID S11B-1	Lymph Nodes_1	59	0.76363636
ID S11B-1	Lymph Nodes_1	106	0.36363636
ID S11B-1	Lymph Nodes_1	181	0.49090909
ID S11B-1	Lymph Nodes_1	227	0.76363636

ID S11B-1	Lymph Nodes_3	0	1
ID S11B-1	Lymph Nodes_3	59	1
ID S11B-1	Lymph Nodes_3	106	1.11111111
ID S11B-1	Lymph Nodes_3	181	1
ID S11B-1	Lymph Nodes_3	227	1
ID S11B-1	Lymph Nodes_4	0	1
ID S11B-1	Lymph Nodes_4	59	0.83333333
ID S11B-1	Lymph Nodes_4	106	0.83333333
ID S11B-1	Lymph Nodes_4	181	1
ID S11B-1	Lymph Nodes_4	227	1
ID S11B-2	Adrenal Glands_2	0	1
ID S11B-2	Adrenal Glands_2	60	0.9375
ID S11B-2	Adrenal Glands_2	151	0.65625
ID S11B-2	Adrenal Glands_2	199	0.75
ID S11B-2	Adrenal Glands_2	246	0.78125
ID S11B-2	Adrenal Glands_2	291	0.78125
ID S11B-2	Adrenal Glands_2	337	0.8125
ID S11B-2	Adrenal Glands_2	379	0.90625
ID S11B-2	Adrenal Glands_3	0	1
ID S11B-2	Adrenal Glands_3	60	0.78947368
ID S11B-2	Adrenal Glands_3	151	0.84210526
ID S11B-2	Adrenal Glands_3	199	0.89473684
ID S11B-2	Adrenal Glands_3	246	0.89473684
ID S11B-2	Adrenal Glands_3	291	0.94736842
ID S11B-2	Adrenal Glands_3	337	0.89473684
ID S11B-2	Adrenal Glands_3	379	0.63157895
ID S11B-2	Lung & Bronchus_1	0	1
ID S11B-2	Lung & Bronchus_1	60	1
ID S11B-2	Lung & Bronchus_1	151	1.1
ID S11B-2	Lung & Bronchus_1	199	1
ID S11B-2	Lung & Bronchus_1	246	1
ID S11B-2	Lung & Bronchus_1	291	0.9
ID S11B-2	Lung & Bronchus_1	337	0.9
ID S11B-2	Lung & Bronchus_1	379	1
ID S11B-3	Abdomen_1	0	1
ID S11B-3	Abdomen_1	51	0.89473684
ID S11B-3	Abdomen_1	101	0.75438596
ID S11B-3	Abdomen_1	154	0.68421053
ID S11B-3	Abdomen_1	196	0.64912281
ID S11B-3	Abdomen_1	245	0.71929825

ID S11B-3	Abdomen_1	289	0.75438596
ID S11B-3	Lymph Nodes_2	0	1
ID S11B-3	Lymph Nodes_2	51	0.86486486
ID S11B-3	Lymph Nodes_2	101	0.78378378
ID S11B-3	Lymph Nodes_2	154	0.78378378
ID S11B-3	Lymph Nodes_2	196	0.78378378
ID S11B-3	Lymph Nodes_2	245	0.83783784
ID S11B-3	Lymph Nodes_2	289	1.02702703
ID S12A-8	Lung & Bronchus_1	0	1
ID S12A-8	Lung & Bronchus_1	42	1.08695652
ID S12A-8	Lung & Bronchus_1	69	1.13043478
ID S12A-8	Lung & Bronchus_2	0	1
ID S12A-8	Lung & Bronchus_2	42	0.76595745
ID S12A-8	Lung & Bronchus_2	69	0.72340426
ID S12A-9	Liver_3	0	1
ID S12A-9	Liver_3	74	0.97959184
ID S12A-9	Liver_3	110	0.97959184
ID S12A-9	Lung & Bronchus_1	0	1
ID S12A-9	Lung & Bronchus_1	74	1.06060606
ID S12A-9	Lung & Bronchus_1	110	1.06060606
ID S12A-9	Lymph Nodes_2	0	1
ID S12A-9	Lymph Nodes_2	74	1
ID S12A-9	Lymph Nodes_2	110	0.76923077
ID S12A-9	Spleen_4	0	1
ID S12A-9	Spleen_4	74	1.14285714
ID S12A-9	Spleen_4	110	1.07142857
ID S12A-1	Lung & Bronchus_1	0	1
ID S12A-1	Lung & Bronchus_1	42	1
ID S12A-1	Lung & Bronchus_1	71	0.94736842
ID S12A-1	Lymph Nodes_2	0	1
ID S12A-1	Lymph Nodes_2	42	0.9375
ID S12A-1	Lymph Nodes_2	71	0.8125
ID S12A-2	Abdomen_2	0	1
ID S12A-2	Abdomen_2	65	0.92857143
ID S12A-2	Abdomen_2	80	0.78571429
ID S12A-2	Adrenal Glands_1	0	1
ID S12A-2	Adrenal Glands_1	65	0.86956522
ID S12A-2	Adrenal Glands_1	80	1
ID S12A-3	Lung & Bronchus_1	0	1
ID S12A-3	Lung & Bronchus_1	49	0.9

ID S12A-3	Lung & Bronchus_1	111	1.03333333
ID S12A-3	Lung & Bronchus_2	0	1
ID S12A-3	Lung & Bronchus_2	49	0.90625
ID S12A-3	Lung & Bronchus_2	111	0.96875
ID S12A-4	Chest_2	0	1
ID S12A-4	Chest_2	37	1
ID S12A-4	Chest_2	64	1
ID S12A-4	Chest_3	0	1
ID S12A-4	Chest_3	37	1
ID S12A-4	Chest_3	64	0.98214286
ID S12A-4	Liver_5	0	1
ID S12A-4	Liver_5	37	1.04477612
ID S12A-4	Liver_5	64	1.08955224
ID S12A-4	Lung & Bronchus_4	0	1
ID S12A-4	Lung & Bronchus_4	37	0.94117647
ID S12A-4	Lung & Bronchus_4	64	0.94117647
ID S12A-5	Lung & Bronchus_1	0	1
ID S12A-5	Lung & Bronchus_1	67	1
ID S12A-5	Lung & Bronchus_1	124	0.96226415
ID S12A-5	Lymph Nodes_2	0	1
ID S12A-5	Lymph Nodes_2	67	1.05
ID S12A-5	Lymph Nodes_2	124	1
ID S12A-5	Lymph Nodes_3	0	1
ID S12A-5	Lymph Nodes_3	67	1
ID S12A-5	Lymph Nodes_3	124	0.89473684
ID S12A-6	Lymph Nodes_1	0	1
ID S12A-6	Lymph Nodes_1	46	0.96153846
ID S12A-6	Lymph Nodes_1	76	0.92307692
ID S12A-6	Lymph Nodes_2	0	1
ID S12A-6	Lymph Nodes_2	46	0.95
ID S12A-6	Lymph Nodes_2	76	1
ID S12A-7	Lung & Bronchus_1	0	1
ID S12A-7	Lung & Bronchus_1	46	0.9375
ID S12A-7	Lung & Bronchus_1	95	1
ID S12A-7	Lung & Bronchus_1	140	1
ID S12A-7	Lung & Bronchus_1	179	1
ID S12A-7	Lung & Bronchus_1	228	1
ID S12A-7	Lung & Bronchus_2	0	1
ID S12A-7	Lung & Bronchus_2	46	0.92592593
ID S12A-7	Lung & Bronchus_2	95	1

ID S12A-7	Lung & Bronchus_2	140	1.14814815
ID S12A-7	Lung & Bronchus_2	179	1
ID S12A-7	Lung & Bronchus_2	228	1.07407407
ID S12B-1	Liver_2	0	1
ID S12B-1	Liver_2	55	0.63636364
ID S12B-1	Liver_2	102	0.95454545
ID S12B-1	Liver_3	0	1
ID S12B-1	Liver_3	55	1
ID S12B-1	Liver_3	102	1.29411765
ID S12B-1	Lung & Bronchus_1	0	1
ID S12B-1	Lung & Bronchus_1	55	0.87179487
ID S12B-1	Lung & Bronchus_1	102	0.97435897
ID S12B-2	Liver_1	0	1
ID S12B-2	Liver_1	79	1.08235294
ID S12B-2	Liver_1	138	1.14117647
ID S12B-2	Liver_2	0	1
ID S12B-2	Liver_2	79	1.23913043
ID S12B-2	Liver_2	138	1.39130435
ID S12B-3	Lung & Bronchus_1	0	1
ID S12B-3	Lung & Bronchus_1	57	1.08974359
ID S12B-3	Lung & Bronchus_1	98	1.23076923
ID S12B-3	Lung & Bronchus_1	140	1.46153846
ID S12B-3	Lymph Nodes_2	0	1
ID S12B-3	Lymph Nodes_2	57	0.86486486
ID S12B-3	Lymph Nodes_2	98	0.97297297
ID S12B-3	Lymph Nodes_2	140	1.24324324
ID S12B-3	Lymph Nodes_3	0	1
ID S12B-3	Lymph Nodes_3	57	0.96296296
ID S12B-3	Lymph Nodes_3	98	1.03703704
ID S12B-3	Lymph Nodes_3	140	1.22222222
ID S12B-4	Lung & Bronchus_1	0	1
ID S12B-4	Lung & Bronchus_1	67	1.07042254
ID S12B-4	Lung & Bronchus_1	123	1.08450704
ID S12B-4	Lung & Bronchus_2	0	1
ID S12B-4	Lung & Bronchus_2	67	1.03030303
ID S12B-4	Lung & Bronchus_2	123	1.28787879
ID S12B-4	Lung & Bronchus_3	0	1
ID S12B-4	Lung & Bronchus_3	67	0.95652174
ID S12B-4	Lung & Bronchus_3	123	1.17391304
ID S12B-5	Lymph Nodes_1	0	1

ID S12B-5	Lymph Nodes_1	27	1
ID S12B-5	Lymph Nodes_1	42	0.53333333
ID S12B-5	Lymph Nodes_1	70	0.53333333
ID S12B-5	Lymph Nodes_2	0	1
ID S12B-5	Lymph Nodes_2	27	0.75
ID S12B-5	Lymph Nodes_2	42	0.625
ID S12B-5	Lymph Nodes_2	70	0.375
ID S12B-6	Adrenal Glands_4	0	1
ID S12B-6	Adrenal Glands_4	63	0.75
ID S12B-6	Adrenal Glands_4	119	1.11111111
ID S12B-6	Lung & Bronchus_1	0	1
ID S12B-6	Lung & Bronchus_1	63	0.65909091
ID S12B-6	Lung & Bronchus_1	119	0.97727273
ID S12B-6	Lung & Bronchus_2	0	1
ID S12B-6	Lung & Bronchus_2	63	0.69565217
ID S12B-6	Lung & Bronchus_2	119	0.7826087
ID S12B-6	Lymph Nodes_3	0	1
ID S12B-6	Lymph Nodes_3	63	0.64705882
ID S12B-6	Lymph Nodes_3	119	1.05882353
ID S9-1	Adrenal Glands_3	0	1
ID S9-1	Adrenal Glands_3	56	1.54545455
ID S9-1	Adrenal Glands_3	86	2.27272727
ID S9-1	Adrenal Glands_3	130	3.63636364
ID S9-1	Kidney_2	0	1
ID S9-1	Kidney_2	56	0.81818182
ID S9-1	Kidney_2	86	0.81818182
ID S9-1	Kidney_2	130	1.13636364
ID S9-1	Lung & Bronchus_1	0	1
ID S9-1	Lung & Bronchus_1	56	0.66197183
ID S9-1	Lung & Bronchus_1	86	0.63380282
ID S9-1	Lung & Bronchus_1	130	1.18309859
ID S9-2	Chest_2	0	1
ID S9-2	Chest_2	80	0.89285714
ID S9-2	Chest_2	136	1.03571429
ID S9-2	Chest_2	189	0.96428571
ID S9-2	Chest_2	227	1.5
ID S9-2	Chest_3	0	1
ID S9-2	Chest_3	80	0.73684211
ID S9-2	Chest_3	136	0.73684211
ID S9-2	Chest_3	189	0.89473684

ID S9-2	Chest_3	227	0.89473684
ID S9-2	Lymph Nodes_1	0	1
ID S9-2	Lymph Nodes_1	80	0.85185185
ID S9-2	Lymph Nodes_1	136	0.85185185
ID S9-2	Lymph Nodes_1	189	0.66666667
ID S9-2	Lymph Nodes_1	227	0.85185185
ID S9-2	Lymph Nodes_4	0	1
ID S9-2	Lymph Nodes_4	80	0.94117647
ID S9-2	Lymph Nodes_4	136	1.11764706
ID S9-2	Lymph Nodes_4	189	1.11764706
ID S9-2	Lymph Nodes_4	227	1.47058824
ID S9-3	Lung & Bronchus_1	0	1
ID S9-3	Lung & Bronchus_1	60	1
ID S9-3	Lung & Bronchus_1	102	0.97222222
ID S9-3	Lung & Bronchus_1	150	0.97222222
ID S9-3	Lung & Bronchus_1	192	1.02777778
ID S9-3	Lung & Bronchus_1	234	1.02777778
ID S9-3	Lung & Bronchus_1	277	1.02777778
ID S9-3	Lung & Bronchus_1	325	1.05555556
ID S9-3	Lung & Bronchus_1	368	1.02777778
ID S9-3	Lung & Bronchus_1	469	0.88888889
ID S9-3	Lung & Bronchus_1	521	0.88888889
ID S9-3	Lung & Bronchus_1	577	0.91666667
ID S9-3	Lung & Bronchus_2	0	1
ID S9-3	Lung & Bronchus_2	60	0.97222222
ID S9-3	Lung & Bronchus_2	102	0.88888889
ID S9-3	Lung & Bronchus_2	150	0.88888889
ID S9-3	Lung & Bronchus_2	192	0.83333333
ID S9-3	Lung & Bronchus_2	234	0.83333333
ID S9-3	Lung & Bronchus_2	277	0.83333333
ID S9-3	Lung & Bronchus_2	325	0.83333333
ID S9-3	Lung & Bronchus_2	368	0.80555556
ID S9-3	Lung & Bronchus_2	469	0.80555556
ID S9-3	Lung & Bronchus_2	521	0.75
ID S9-3	Lung & Bronchus_2	577	0.75
ID S9-3	Lymph Nodes_3	0	1
ID S9-3	Lymph Nodes_3	60	0.6
ID S9-3	Lymph Nodes_3	102	0.73333333
ID S9-3	Lymph Nodes_3	150	0.86666667
ID S9-3	Lymph Nodes_3	192	0.46666667

ID S9-3	Lymph Nodes_3	234	0.53333333
ID S9-3	Lymph Nodes_3	277	0.66666667
ID S9-3	Lymph Nodes_3	325	0.73333333
ID S9-3	Lymph Nodes_3	368	0.73333333
ID S9-3	Lymph Nodes_3	469	0.8
ID S9-3	Lymph Nodes_3	521	0.86666667
ID S9-3	Lymph Nodes_3	577	1.2
ID1	Lung & Bronchus_1	0	1
ID1	Lung & Bronchus_1	39	0.91428571
ID1	Lung & Bronchus_1	86	0.68571429
ID1	Lung & Bronchus_1	121	0.62857143
ID1	Lung & Bronchus_2	0	1
ID1	Lung & Bronchus_2	39	1.2
ID1	Lung & Bronchus_2	86	1.2
ID1	Lung & Bronchus_2	121	1.4
ID1	Lung & Bronchus_3	0	1
ID1	Lung & Bronchus_3	39	1
ID1	Lung & Bronchus_3	86	1.0625
ID1	Lung & Bronchus_3	121	1.375
ID2	Lung & Bronchus_1	0	1
ID2	Lung & Bronchus_1	82	0.95652174
ID2	Lung & Bronchus_1	138	1.32608696
ID2	Lymph Nodes_2	0	1
ID2	Lymph Nodes_2	82	0.75
ID2	Lymph Nodes_2	138	0.61363636
ID2	Lymph Nodes_3	0	1
ID2	Lymph Nodes_3	82	1.57142857
ID2	Lymph Nodes_3	138	1.76190476
ID3	Chest_1	0	1
ID3	Chest_1	43	0.58333333
ID3	Chest_1	67	0.25
ID3	Chest_1	114	0.16666667
ID3	Chest_1	155	0.16666667
ID3	Chest_1	196	0.16666667
ID3	Chest_1	245	0.08333333
ID3	Chest_1	289	0.08333333
ID3	Chest_1	331	0.125
ID3	Chest_1	368	0.16666667
ID3	Chest_2	0	1
ID3	Chest_2	43	0.25

ID3	Chest_2	67	0.25
ID3	Chest_2	114	0.1875
ID3	Chest_2	155	0.1875
ID3	Chest_2	196	0.1875
ID3	Chest_2	245	0.25
ID3	Chest_2	289	0.25
ID3	Chest_2	331	0.4375
ID3	Chest_2	368	0.4375
ID3	Chest_3	0	1
ID3	Chest_3	43	1.375
ID3	Chest_3	67	0.875
ID3	Chest_3	114	0.4375
ID3	Chest_3	155	0.375
ID3	Chest_3	196	0.3125
ID3	Chest_3	245	0.3125
ID3	Chest_3	289	0.25
ID3	Chest_3	331	0.25
ID3	Chest_3	368	0.3125
ID3	Chest_4	0	1
ID3	Chest_4	43	1.66666667
ID3	Chest_4	67	0.91666667
ID3	Chest_4	114	0.66666667
ID3	Chest_4	155	0.66666667
ID3	Chest_4	196	0.58333333
ID3	Chest_4	245	0.5
ID3	Chest_4	289	0.41666667
ID3	Chest_4	331	0.41666667
ID3	Chest_4	368	0.5
ID4	Chest_1	0	1
ID4	Chest_1	35	1
ID4	Chest_1	59	0.88
ID4	Chest_1	106	0.96
ID4	Chest_1	148	0.84
ID4	Chest_1	191	0.76
ID4	Chest_1	242	0.76
ID4	Chest_1	288	0.68
ID4	Chest_1	332	0.44
ID4	Chest_1	367	0.44
ID4	Chest_1	413	0.48
ID4	Chest_1	463	0.48

ID4	Chest_1	504	0.48
ID4	Chest_1	548	0.52
ID4	Chest_1	584	0.56
ID4	Chest_2	0	1
ID4	Chest_2	35	1.36363636
ID4	Chest_2	59	0.63636364
ID4	Chest_2	106	0
ID4	Chest_2	148	0
ID4	Chest_2	191	0
ID4	Chest_2	242	0
ID4	Chest_2	288	0
ID4	Chest_2	332	0
ID4	Chest_2	367	0
ID4	Chest_2	413	0
ID4	Chest_2	463	0
ID4	Chest_2	504	0
ID4	Chest_2	548	0
ID4	Chest_2	584	0
ID4	Chest_3	0	1
ID4	Chest_3	35	0.76190476
ID4	Chest_3	59	0.19047619
ID4	Chest_3	106	0
ID4	Chest_3	148	0
ID4	Chest_3	191	0
ID4	Chest_3	242	0
ID4	Chest_3	288	0
ID4	Chest_3	332	0
ID4	Chest_3	367	0
ID4	Chest_3	413	0
ID4	Chest_3	463	0
ID4	Chest_3	504	0
ID4	Chest_3	548	0
ID4	Chest_3	584	0
ID4	Chest_4	0	1
ID4	Chest_4	35	1.19047619
ID4	Chest_4	59	0.42857143
ID4	Chest_4	106	0
ID4	Chest_4	148	0
ID4	Chest_4	191	0
ID4	Chest_4	242	0

ID4	Chest_4	288	0
ID4	Chest_4	332	0
ID4	Chest_4	367	0
ID4	Chest_4	413	0
ID4	Chest_4	463	0
ID4	Chest_4	504	0
ID4	Chest_4	548	0
ID4	Chest_4	584	0

RECIST sum ratio

id	time	tumor_ratio
ID S10A-1	0	1
ID S10A-1	48	1.13178295
ID S10A-1	73	1.06976744
ID S10A-2	0	1
ID S10A-2	56	1.03030303
ID S10A-2	112	1.18181818
ID S10A-3	0	1
ID S10A-3	62	0.96590909
ID S10A-3	156	1.04545455
ID S10A-3	202	1.10227273
ID S10A-3	244	1.125
ID S10A-3	286	1.10227273
ID S10A-3	328	1.11363636
ID S10B-1	0	1
ID S10B-1	69	1.13043478
ID S10B-1	125	1.61956522
ID S10B-2	0	1
ID S10B-2	71	1.02459016
ID S10B-2	126	1.09836066
ID S10B-2	210	1.27868853
ID S10B-3	0	1
ID S10B-3	54	1.15942029
ID S10B-3	96	1.17391304
ID S10B-3	141	1.49275362
ID S10B-4	0	1
ID S10B-4	59	1.15116279
ID S10B-4	122	1.02325581
ID S10B-5	0	1
ID S10B-5	50	1.11320755
ID S10B-5	75	1.18867925
ID S10B-6	0	1
ID S10B-6	64	1
ID S10B-6	123	1.09090909
ID S10B-6	176	1.15151515
ID S10B-6	229	1.12121212
ID S11A-1	0	1

ID S11A-1	61	0.82835821
ID S11A-1	100	0.70895522
ID S11A-1	142	0.68656716
ID S11A-2	0	1
ID S11A-2	56	0.87037037
ID S11A-2	112	0.7962963
ID S11A-2	166	0.7962963
ID S11B-1	0	1
ID S11B-1	59	0.85606061
ID S11B-1	106	0.67424242
ID S11B-1	181	0.73484849
ID S11B-1	227	0.88636364
ID S11B-2	0	1
ID S11B-2	60	0.90163934
ID S11B-2	151	0.78688525
ID S11B-2	199	0.83606557
ID S11B-2	246	0.85245902
ID S11B-2	291	0.85245902
ID S11B-2	337	0.85245902
ID S11B-2	379	0.83606557
ID S11B-3	0	1
ID S11B-3	51	0.88297872
ID S11B-3	101	0.76595745
ID S11B-3	154	0.72340426
ID S11B-3	196	0.70212766
ID S11B-3	245	0.76595745
ID S11B-3	289	0.86170213
ID S12A-8	0	1
ID S12A-8	42	0.87142857
ID S12A-8	69	0.85714286
ID S12A-9	0	1
ID S12A-9	74	1.03521127
ID S12A-9	110	1.00704225
ID S12A-1	0	1
ID S12A-1	42	0.98148148
ID S12A-1	71	0.90740741
ID S12A-2	0	1
ID S12A-2	65	0.89189189
ID S12A-2	80	0.91891892
ID S12A-3	0	1

ID S12A-3	49	0.90217391
ID S12A-3	111	1.01086957
ID S12A-4	0	1
ID S12A-4	37	1.00456621
ID S12A-4	64	1.01369863
ID S12A-5	0	1
ID S12A-5	67	1.01086957
ID S12A-5	124	0.95652174
ID S12A-6	0	1
ID S12A-6	46	0.95652174
ID S12A-6	76	0.95652174
ID S12A-7	0	1
ID S12A-7	46	0.93023256
ID S12A-7	95	1
ID S12A-7	140	1.09302326
ID S12A-7	179	1
ID S12A-7	228	1.04651163
ID S12B-1	0	1
ID S12B-1	55	0.8631579
ID S12B-1	102	1.08421053
ID S12B-2	0	1
ID S12B-2	79	1.13740458
ID S12B-2	138	1.22900763
ID S12B-3	0	1
ID S12B-3	57	1.00704225
ID S12B-3	98	1.12676056
ID S12B-3	140	1.35915493
ID S12B-4	0	1
ID S12B-4	67	1.0375
ID S12B-4	123	1.18125
ID S12B-5	0	1
ID S12B-5	27	0.87096774
ID S12B-5	42	0.58064516
ID S12B-5	70	0.4516129
ID S12B-6	0	1
ID S12B-6	63	0.69166667
ID S12B-6	119	0.99166667
ID S9-1	0	1
ID S9-1	56	0.78846154
ID S9-1	86	0.84615385

ID S9-1	130	1.43269231
ID S9-2	0	1
ID S9-2	80	0.85714286
ID S9-2	136	0.93406593
ID S9-2	189	0.89010989
ID S9-2	227	1.17582418
ID S9-3	0	1
ID S9-3	60	0.91954023
ID S9-3	102	0.89655172
ID S9-3	150	0.91954023
ID S9-3	192	0.85057471
ID S9-3	234	0.86206897
ID S9-3	277	0.88505747
ID S9-3	325	0.90804598
ID S9-3	368	0.88505747
ID S9-3	469	0.83908046
ID S9-3	521	0.82758621
ID S9-3	577	0.89655172
ID1	0	1
ID1	39	1
ID1	86	0.89393939
ID1	121	0.98484849
ID2	0	1
ID2	82	0.99099099
ID2	138	1.12612613
ID3	0	1
ID3	43	0.88235294
ID3	67	0.51470588
ID3	114	0.32352941
ID3	155	0.30882353
ID3	196	0.27941177
ID3	245	0.25
ID3	289	0.22058824
ID3	331	0.27941177
ID3	368	0.32352941
ID4	0	1
ID4	35	1.03846154
ID4	59	0.53846154
ID4	106	0.30769231
ID4	148	0.26923077

ID4	191	0.24358974
ID4	242	0.24358974
ID4	288	0.21794872
ID4	332	0.14102564
ID4	367	0.14102564
ID4	413	0.15384615
ID4	463	0.15384615
ID4	504	0.15384615
ID4	548	0.16666667
ID4	584	0.17948718
